# Modeling the Influence of Ion Channels on Neuron Dynamics in *Drosophila*

**DOI:** 10.3389/fncom.2015.00139

**Published:** 2015-11-18

**Authors:** Sandra D. Berger, Sharon M. Crook

**Affiliations:** ^1^School of Life Sciences, Arizona State UniversityTempe, AZ, USA; ^2^School of Mathematical and Statistical Sciences, Arizona State UniversityTempe, AZ, USA

**Keywords:** membrane excitability, ion channel kinetics, *Drosophila* model, neuronal dynamics, bifurcation studies

## Abstract

Voltage gated ion channels play a major role in determining a neuron's firing behavior, resulting in the specific processing of synaptic input patterns. *Drosophila* and other invertebrates provide valuable model systems for investigating ion channel kinetics and their impact on firing properties. Despite the increasing importance of *Drosophila* as a model system, few computational models of its ion channel kinetics have been developed. In this study, experimentally observed biophysical properties of voltage gated ion channels from the fruitfly *Drosophila melanogaster* are used to develop a minimal, conductance based neuron model. We investigate the impact of the densities of these channels on the excitability of the model neuron. Changing the channel densities reproduces different *in situ* observed firing patterns and induces a switch from integrator to resonator properties. Further, we analyze the preference to input frequency and how it depends on the channel densities and the resulting bifurcation type the system undergoes. An extension to a three dimensional model demonstrates that the inactivation kinetics of the sodium channels play an important role, allowing for firing patterns with a delayed first spike and subsequent high frequency firing as often observed in invertebrates, without altering the kinetics of the delayed rectifier current.

## 1. Introduction

It is well-known that different neuron types exhibit distinct characteristic features under standardized or similar conditions such as constant current injection, due in part to the influence of differing ion channel kinetics and distributions (Shepherd, 2004). Experimental and theoretical studies show that differences in spiking patterns can be related to different combinations of ion channel densities (Goldman et al., 2001; Zeberg et al., 2010) with different channel kinetics. These differences affect the timing of action potentials (APs) and influence subthreshold integration of synaptic input and the filtering properties of the neuronal structure, resulting in bandpass or highpass filtering properties. Consequently, the response of a neuron to synaptic input depends on the underlying dynamics of membrane excitability. Hodgkin classified neurons according to their spiking behavior upon steady current injection, and the resulting frequency–current relationships (*f* –*I* curves) generally can be divided into three distinct classes (Hodgkin, 1948). Numerous subsequent studies have analyzed the relationship between this classification and the output properties of a model neuron (Ermentrout, 1996; Rinzel and Ermentrout, 1998; Gutkin et al., 2003; St-Hilaire and Longtin, 2004; Tateno et al., 2004; Tateno and Robinson, 2006, 2007) and, from a dynamical systems point of view, how this classification relates to the underlying mathematical structure of the model (Izhikevich, 2007; Prescott et al., 2008).

In spite of these theoretical studies, the impact of specific ion channel kinetics on neuronal function remains largely unclear. *Drosophila* provides a valuable model system for investigating ion channel kinetics and their impact on firing properties. Neurons can be identified individually, and many molecular mechanisms are comparable to those in vertebrate systems. The spiking responses and neuronal morphology in these neurons have been investigated at different developmental stages (Choi et al., 2004) and changes in these properties have been observed during development (Duch and Levine, 2000), after targeted genetic manipulations, and under different pharmacological conditions (Peng and Wu, 2007; Duch et al., 2008; Ryglewski and Duch, 2009). In particular, the use of *Drosophila* in research is of great interest due to the development of new genetic tools for experimentation. Despite the increasing importance of *Drosophila* as a model system (reviewed by Baines and Pym, 2006 and Corty et al., 2009), few computational models of its ion channels have been developed. The use of computational modeling techniques can help predict the behavior of membrane dynamics at experimentally inaccessible locations and help connect electrophysiological and other molecular biological findings to neuronal function. Here, we create mathematical models, based on experimental data from *Drosophila*, in order to conduct a computational study that investigates how changes of different parameters in physiological ranges influence the input-output properties of neurons. We focus on the identified *Drosophila* motoneuron 5 (MN5) since its morphology, electrophysiology and certain aspects of its behavior during flight have been well-characterized experimentally.

The generation of action potentials, along with their shape and firing patterns, depends in large part on voltage gated sodium (Na^+^) and potassium (K^+^) channels. *Drosophila* has only one confirmed Na^+^ channel gene *DmNa*_*v*_ (Miyazaki et al., 1996; Mee et al., 2004), which is subject to alternative splicing. The voltage dependence of the macroscopic currents carried by the different splice variants has been characterized in heterologous expression systems using voltage clamp recordings (Olson et al., 2008; Lin et al., 2009). K^+^ channels show the greatest diversity among ion channels (Jan et al., 1977; Coetzee et al., 1999), where voltage gated ion channels fall roughly into two categories, the non-inactivating or slowly inactivating delayed rectifier and the rapidly inactivating, transient A-type currents (Hille, 1992). In *Drosophila* neurons two genes, *Shab* and *Shaw*, encode delayed rectifier current conducting channels. Shab and Shaw channels are members of the K_*v*_2 and K_*v*_3 subfamilies, respectively (Wei et al., 1990; Covarrubias et al., 1991; Tsunoda and Salkoff, 1995a,b). Shaw channels demonstrate low voltage sensitivity, suggesting that they operate as leak channels, while Shab channels conduct the majority of delayed rectifier currents (Tsunoda and Salkoff, 1995b). The kinetics of the Shab channel are reported to be comparable to the classical (as described by Hodgkin–Huxley) delayed rectifier K^+^ channel (Tsunoda and Salkoff, 1995b). Two further genes encode channels conducting A-type currents that show fast inactivation kinetics. Activation and inactivation properties of *Drosophila* voltage gated K^+^ channels have been characterized in homologous expression systems as well as in *Drosophila* neurons (Covarrubias et al., 1991; Islas and Sigworth, 1999; Tsunoda and Salkoff, 1995b; Gasque et al., 2005). Their contributions to firing properties have been studied using pharmacology and genetical manipulations in order to remove the currents (Choi et al., 2004; Gasque et al., 2005; Peng and Wu, 2007; Ryglewski and Duch, 2009; Ping et al., 2011). Comparing different mutant neurons indicates that Shab is required for repetitive spiking, while A-type channels regulate the firing frequency and lower the spike threshold to induce a delay to first spike (Choi et al., 2004; Ping et al., 2011).

**Figure 1:**
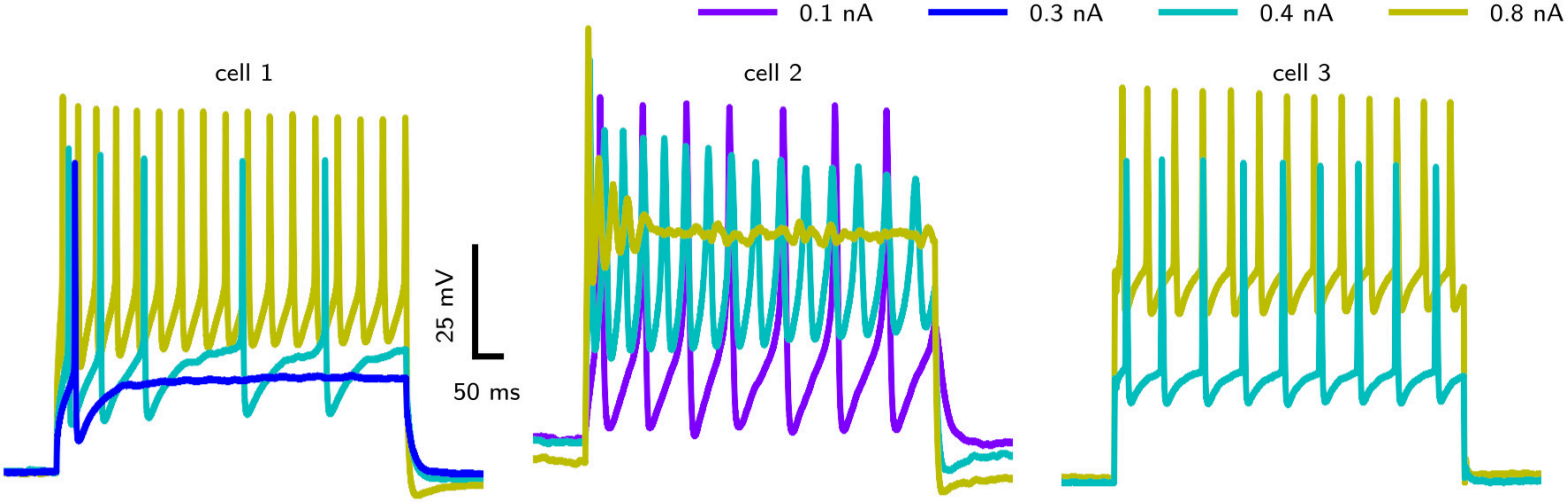
**Different firing behaviors from three ***Drosophila*** MN5s**. Intracellular recordings in whole cell patch configuration carried out by S. Ryglewski in the Duch laboratory reveal substantial animal to animal variation in the responses to current pulses (*n* = 52). Cell 1 (38 %): A single action potential for a low-amplitude current stimulation, with repetitive spiking that adapts in frequency for slightly increased stimulation. Firing frequency increases with stimulation amplitude. Cell 2 (8 %): A very low-amplitude stimulation produces repetitive spiking with a relatively large duration of single APs. Stimulations with an amplitude that is close to the firing threshold observed in the other cases (0.4 nA) induces a large initial spike with subsequent broader spikes that diminish in amplitude. Increasing the stimulation amplitude further results in dampening oscillations that finalize in a depolarization block. Cell 3 (42 %): Repetitive firing is induced by a stimulation amplitude of 0.4 nA, while the cell remains quiescent with stimulation amplitudes of 0.3 nA. Higher stimulus amplitudes result in only a slight increase in firing frequency (42 %). Experimental methods provided by Ryglewski and Duch (2009).

However, accessing the specific role of different ion channels is challenged by the considerable amount of variability across individuals. *Drosophila* MN5 shows considerable animal to animal variation in spiking behaviors upon current injection (Duch et al., 2008), ranging from non-repetitive responses (single spikes or single graded responses) to repetitive spiking with different *f* –*I* curves and varying delays to first spike. Data from intracellular recordings in whole cell patch configuration (shown in Figure 1) demonstrate different firing behaviors seen in *Drosophila* MN5. Some cells exhibit a single AP with no delay to first spike for small *I*_*app*_ or display only a slight increase in firing frequency with increasing *I*_*app*_. This indicates a discontinuous *f* –*I* curve and relates to a dynamical system close to a Hopf bifurcation (type II dynamics). Other cells have a comparably lower firing threshold, where smaller *I*_*app*_-values induce low frequency spiking. In this case a continuous *f* –*I* curve is possible, which can be related to a saddle node on invariant cycle (SNIC) bifurcation (type I dynamics). In rare cases, repetitive spiking occurs after a long delay, where the interspike intervall (ISI) is smaller than the initial delay and higher amplitudes elicit higher frequency spiking without initial delay (see Herrera-Valdez et al., 2013). In dynamical systems this type of behavior is observed if the system is close to a saddle small homoclinic bifurcation (Izhikevich, 2007).

In addition to animal to animal variability, compensation mechanisms and modulation adjust the membrane properties of neurons, and experimental studies using genetic knock downs can be compromised by these homeostatic regulation mechanisms (Marder, 2011). Here we determine whether this known variability can be captured sufficiently by a minimal model with only two realistic channel types. This provides a foundation for understanding the roles of these channels and for future studies with additional channel types where detailed mathematical analysis is not possible due to the large number of variables. We begin with a model of a patch of excitable membrane where we carefully develop models of Na^+^ and K^+^ voltage gated ion channels displaying kinetics based on channels expressed in *Drosophila*. The Na^+^ channel is based on kinetics of DmNa_V_29, which mediates a fast inward current, and the K^+^ channel is based on kinetics of Shab, which mediates a delayed rectifier outward current. In contrast to the electro-diffusion based model in our previous work (Herrera-Valdez et al., 2013), here we use a conductance-based model, where the parameters of the model are adjusted to better resemble published data. First, using a mathematically reduced two dimensional model, we ask whether changing the density of Shab channels is sufficient to switch between bifurcation types and whether this can cause the different observed response properties. Employing sinusoidal current stimulation, we confirm the occurrence of qualitatively different responses to rhythmic synaptic input. Next, we extend the model to three dimensions and find that adjusting the inactivation kinetics of Na^+^ promotes a saddle small homoclinic bifurcation leading to a delayed first spike, without changing the kinetics of the Shab channels.

## 2. Materials and methods

### 2.1. Conductance based membrane model

Membrane potential (*V*_*m*_) dynamics are described with a Hodgkin–Huxley type conductance based model (Hodgkin and Huxley, 1952), where the current balance equation takes the form

(1) $$\begin{array}{l} c_{m}\frac{dV_{m}\left(t\right)}{dt}=-\underset{i}{\sum }I_{i}+I_{app}. \end{array}$$

Here *c*_*m*_ is the membrane capacitance, *I*_*app*_ the applied current, and *I*_*i*_ is the ionic current mediated by channel type *i*. The value of *c*_*m*_ was determined using the whole cell capacitance (≈1.3*nF*) as measured in voltage clamp (Ryglewski and Duch, 2009) and the surface area of MN5 (≈10, 000μ*m*^2^) as determined by geometric reconstruction from confocal image stacks (Vonhoff and Duch, 2010). *I*_*i*_ are modeled according to Ohm's law:

(2) $$\begin{array}{l} I_{i}=g_{i}\left(V_{m}-V_{i}\right), \end{array}$$

where *g*_*i*_ is the voltage dependent conductance and *V*_*i*_ is the reversal potential of channel *i*. The general formulation for *g*_*i*_ includes two gating variables representing activation and inactivation kinetics, *p* and *q*, respectively. Each gating variable is associated with a number of independently operating gates *j* and *k*, respectively; such that

(3) $$\begin{array}{l} g_{i}={\bar{g}}_{i}p_{i}^{j}q_{i}^{k}, \end{array}$$

where ${\bar{g}}_{i}$ is the maximal conductance of channel type *i*. The open probability of a gate *p* is described with

(4) $$\begin{array}{l} \frac{dp}{dt}=\frac{p_{\infty }\left(V_{m}\right)-p}{\tau_{p}\left(V_{m}\right)}, \end{array}$$

where *p*_∞_ is the steady state function and τ_*p*_ is the voltage dependent change of the time constant. The description of gate *q* takes the same form. By assuming that opening or closing of a gate results from charged particles moving across the membrane in response to an electric field, *p*_∞_ and τ_*p*_ become

(5) $$\begin{array}{r} p_{\infty }\left(V_{m}\right)=\frac{1}{1+\exp\left(\frac{-z_{p}e\left(V_{m}\;-\;V_{p}^{H}\right)}{k_{B}T}\right)}and\;\\ \tau_{p}\left(V_{m}\right)=\frac{\exp\left(\frac{-\gamma_{p}z_{p}e\left(V_{m}\;-\;V_{p}^{H}\right)}{k_{B}T}\right)}{r_{p}\left(1+\exp\left(\frac{z_{p}e\left(V_{m}\;-\;V_{p}^{H}\right)}{k_{B}T}\right)\right)}, \end{array}$$

where $k_{B}\approx 1.3806582\times 10^{-23}$ Jk^−1^ is the Boltzmann constant, *T* = 295.15 K the temperature, $V_{p}^{H}$ the half activation voltage, *r*_*p*_ the rate of activation, and γ_*p*_ the relative position of the energy barrier for a gating particle in the membrane. *z*_*p*_*e* is the gating charge, where *e* ≈ 1.60217733 × 10^−19^C is the elementary charge and *z*_*p*_ reflects the amount and distance the gating particle is moved (Willms et al., 1999; Destexhe and Huguenard, 2000). The parameter values are summarized in Table 1.

**Table 1:** **Parameters used in the models**.

**Name (unit)**	**Description**	**Value**
*c*_*m*_ (nF)	Membrane capacitance	0.13
*V*_*L*_ (mV)	Reversal potential of the leak current	–60
*V*_*K*_ (mV)	Reversal potential of the K^+^ current	–72
*V*_*Na*_ (mV)	Reversal potential of the Na^+^ current	55
${\bar{g}}_{Na}$ (µS)	Maximal conductance of DmNa_*v*_ channels	15.62
${\tilde{g}}_{L}$	Ratio of the maximal conductance of leak and DmNa_*v*_ channels	0.036

*Excluding parameters for ion channel kinetics*.

### 2.2. Minimal ion channel model

For the minimal model, one Na^+^ current, one K^+^ current, and one leak current determine the intrinsic dynamics of *V*_*m*_. The voltage dependence of the Na^+^ current (*I*_*Na*_) is based on currents mediated by channels encoded by the splice variant *DmNa*_*v*_*29*, which is among others expressed in adult *Drosophila* neurons. The K^+^ current (*I*_*sb*_) is a delayed rectifier current based on kinetics reported for Shab channels, where we neglect the slow inactivation of those channels to aid with mathematical analysis. The leak current, *I*_*Leak*_, accounts for currents with relatively small voltage dependence. Generally, the membrane currents for Na^+^ and K^+^ and the leak current take the form

(6) $$\begin{array}{lll} I_{Na} & ={\bar{g}}_{Na}m^{3}h\left(V_{m}-V_{Na}\right),\; & \; \end{array}$$

(7) $$\begin{array}{lll} I_{sb} & ={\bar{g}}_{sb}b^{4}\left(V_{m}-V_{K}\right),\; & \; \end{array}$$

(8) $$\begin{array}{lll} I_{Leak} & ={\bar{g}}_{L}\left(V_{m}-V_{L}\right);\; & \; \end{array}$$

however, we consider several variations on this model in order determine and understand the range of cell dynamic behaviors while fitting the parameters to experimental data.

To begin, the fast activation is assumed to be at steady state, and we assume that the inactivation kinetics can be represented as a function of the activation kinetics of the Shab channel gating (Rinzel, 1985; Av-Ron et al., 1991) in order to reduce the model to two dimensions. Further discussion of these assumptions is provided below. This reduced system takes the form

(9) $$I_{Na}={\bar{g}}_{Na}m_{\infty }^{3}(1-b)(V_{m}-V_{Na}),$$

(10) $$\begin{array}{lll} I_{sb} & ={\bar{g}}_{sb}b^{4}\left(V_{m}-V_{K}\right),\; & \; \end{array}$$

(11) $$\begin{array}{lll} I_{Leak} & ={\bar{g}}_{L}\left(V_{m}-V_{L}\right),\; & \; \end{array}$$

allowing for phase plane analysis. Voltage clamp recordings are used to constrain the parameters. When the voltage is held constant at *V*_*cl*_, the solution to Equation (4) is

(12) $$\begin{array}{lll} p\left(t\right) & =p_{\infty }\left(V_{cl}\right)+\left(p_{0}-p_{\infty }\left(V_{cl}\right)\right)\exp\left(\frac{-t}{\tau_{p}\left(V_{cl}\right)}\right);\; & \; \end{array}$$

therefore, the membrane current mediated by a channel denoted by the index *i* becomes

(13) $$\begin{array}{l} I_{i}={\bar{g}}_{i}{\left(p_{\infty }\left(V_{cl}\right)+\left(p_{0}-p_{\infty }\left(V_{cl}\right)\right)\exp\left(\frac{-t}{\tau_{p}\left(V_{cl}\right)}\right)\right)}^{j}\\ {\left(q_{\infty }\left(V_{cl}\right)+\left(q_{0}-q_{\infty }\left(V_{cl}\right)\right)\exp\left(\frac{-t}{\tau_{p}\left(V_{cl}\right)}\right)\right)}^{k}\left(V_{cl}-V_{i}\right). \end{array}$$

To model the activation gating (*m*) of the DmNa_V_29 channel, we use previously published parameters (Herrera-Valdez et al., 2010). Recall that, initially, the parameters for the inactivation gating (*h*) are the same as the parameters for the activation gating (*b*) of the Shab channels. The activation and inactivation curves as reported by Olson et al. (2008) were used to evaluate the channel model. The experimental Na^+^ activation curve was obtained by clamping the cell at a holding potential of −120 mV and recording the current in response to step potentials from −120 to 60 mV in 5 mV increments. The peak currents for each step potential are divided by (*V*_*m*_ − *V*_*Na*_) to obtain the peak conductance. In experiments, Olson et al. (2008) obtained the steady state inactivation curve for Na^+^ using pre-steps from −120 to 40 mV followed by a step potential of −5 mV. The pre-step potential is assumed to be sufficiently low so that essentially all inactivation gates (*h*) are open and all activation gates (*m*) are closed. The duration of the pre-step is assumed to be long enough that the system is at steady state. The peak current of each trace is divided by the maximal peak current among all steps, which is the one in response to the most hyperpolarized pre-step and assumed to be the current amplitude, given all channels are in the open state. We simulate those experiments using Equation (13) for comparison. Subsequently, we incorporate independent Na^+^ inactivation by restoring the third gating variable, *h* using parameters reported for inactivation. Again we simulate the experiments using Equation (13) for comparison of the activation and inactivation curves.

The parameters for *I*_*sb*_ were constrained using voltage clamp recordings from *Drosophila* embryonal cell cultures (Tsunoda and Salkoff, 1995b). Cells were clamped to a holding potential of −50 mV, and the current in response to step potentials from −20 to 50 mV in 10 mV increments was recorded. The published traces were digitized and fitted simultaneously to Equation (13) using a non-linear least-squares optimization algorithm. Different initial conditions were used, that converged all to the same values.

Maximal conductances ${\bar{g}}_{Na}$ and ${\bar{g}}_{sb}$ can be seen as the combination of the amount of channels in the membrane and the maximal conductance of the corresponding single channels. These values were also fitted but since the number of channels can be very different in different neurons, in what follows we focus on the relative amounts of these values.

### 2.3. Software

Bifurcation diagrams were generated with numerical continuation methods, using PyCont a sub-package of PyDSTool (Clewley et al., 2007), which provides an interface to AUTO (Doedel et al., 2000). Phase response curves (PRCs) were calculated with the adjoint method implemented in PyDSTool. Stable and unstable manifolds for saddle points were obtained using XPPAUT (Ermentrout, 2002) with a time step of 0.001 ms. The phase responses were determined by calculating the system's adjoint (Ermentrout and Kopell, 1991) using PyDSTool. Numerical simulations were performed using Python 2.7. The systems of ordinary differential equations are integrated using odeint with default settings from the scipy package, which uses lsoda from the FORTRAN odepac. For nonstiff problems, AdamsĂŹ method was used, and for stiff problems, a method based on backward differentiation formulas was used. Results are compared to results obtained with XPPAUT using time steps of 0.05 and 0.01 ms to ensure accuracy. Fitting of the electrophysiological data was performed using leastsq from the scipy optimization package.

## 3. Results

### 3.1. Fitting of ion channel models

**Figure 2:**
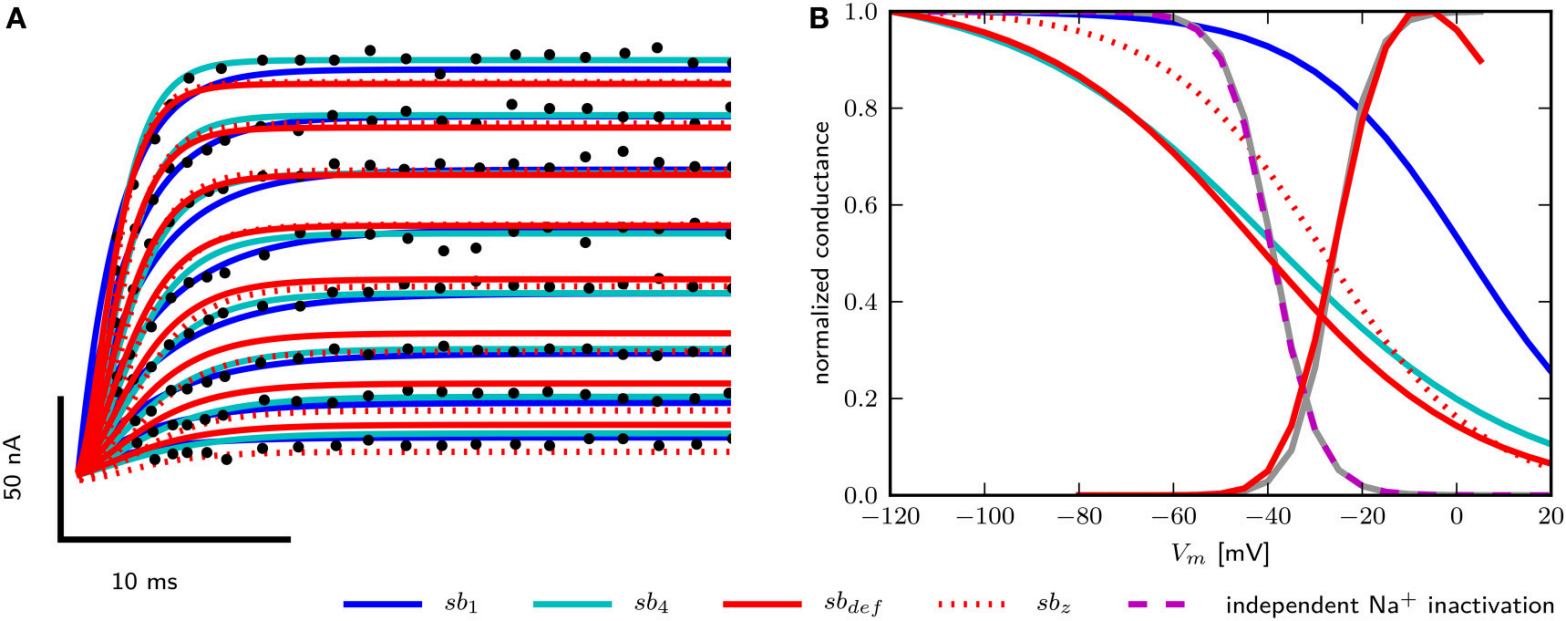
**Data fits of Shab and DmNa_v_29 channel parameters**. **(A)** Digitized points from Figure 4A from Tsunoda and Salkoff (1995b) are marked as black circles. Model of Shab channels with parameters obtained from fits with power one (*sb*_1_), power four (*sb*_4_), hand tuned parameters (*sb*_*def*_) and fits with power four and fixed *z*_*b*_ (*sb*_*z*_). Compare with Figure 7 from Herrera-Valdez et al. (2013). **(B)** Steady state activation and inactivation curves of DmNa_v_29 according to Olson et al. (2008) (gray) and from models (note that the activation kinetics are the same in all models). Results using parameter sets *sb*_1_, *sb*_4_, *sb*_*def*_, and independent inactivation used in the three dimensional model.

To obtain models for Shab channels, traces from voltage clamp recordings (Tsunoda and Salkoff, 1995b) are fit with Equation (13) as described above. The number of gates are usually chosen to best represent the experimentally measured ion current dynamics. Since the inactivation of Shab channels is small, we set the number of inactivation gates to zero as shown in the Section 2 above. For the activation gating (*b*) we use a power of four as used by Hodgkin and Huxley (1952), but also investigate a power of one as in Herrera-Valdez et al. (2013). Both powers provide fits that are in good agreement with the voltage clamp recordings of Shab currents (Figure 2A) although they require different values for other parameters in order to best fit the data. The values of the different parameter sets are summarized in Table 2. In Equation (13) the electromotive force is based on Ohms's law, as it is commonly described in Hodgkin–Huxley type conductance based models and displays a linear current–voltage relationship.

**Table 2:** **Parameter sets for ***I_sb_*****.

**Name**	**Description**	***sb_pup_* (Published data^a^)**	***sb*_1_ (Fits with power one)**	***sb*_4_ (Fits with power four)**	***sb_def_* (Hand tuned from *sb*_4_)**	***sb_z_* (Fits with power four and fixed *z_b_*)**
$V_{b}^{H}$ (mV)	Half-maximum activation voltage for *b* gate	–1.05± 1.20	2.20	–39.3	–42.1	–28.1
*z*_*b*_	Movement of gate *b* gating particle	1.59 ± 0.10	1.51	0.94	1.1	1.5
*r*_*b*_ (ms^−1^)	Rate of activation for *b* gate	–	0.14	0.17	0.2	0.26
γ_*b*_	Position of the energy barrier for *b* gate	–	0.46	0.49	0.38	0.3
Number of gates	Number of independent *b* gates	1	1	4	4	4

*Comparison of published values, fits of digitized experimental data with Equation (13), and hand tuned parameters*.

a *Tsunoda and Salkoff (1995b)*.

For Na^+^ channels, only the parameters for curves used to fit the peak and tail currents from voltage clamp experiments are available (Olson et al., 2008). Therefore, only *z*_*m*_, *z*_*h*_, $V_{m}^{H}$, and $V_{h}^{H}$ can be assessed. For the two dimensional reduced model where the inactivation curve for *I*_*Na*_ depends on the activation of the Shab channels, inactivation is represented best using the term (1−*b*) with a power of one for the Shab gating variable (*b*) (Table 2, Figure 2B) because the half activation of a single gate is close to the value reported for DmNa_v_29 (Table 3). The half inactivation values for other DmNa_v_ splice variants are between −34.9 and −66.4 mV (Olson et al., 2008; Lin et al., 2009), where none of these reported splice variants has a half inactivation that is close to the value found for Shab channel activation using a power of one. In addition, the slope of the resulting inactivation curve for *I*_*Na*_ is too flat, indicating that the gating charge *z*_*b*_ is too small. The Shab channel kinetics were adjusted in an attempt to improve *z*_*b*_, leading to parameter set *sb*_*def*_; however, only a slight improvement could be achieved without compromising the Shab kinetics (Figure 2B). Due to these issues, after analyzing the two dimensional model, we extend the model to three dimensions by restoring the variable *h* for *I*_*Na*_ inactivation, which allows for different parameters for Na^+^ inactivation and K^+^ activation. Adjusting the gating parameter as summarized in Table 2 leads to a good agreement between model and data (Figure 2B) for the three dimensional system.

**Table 3:** **Parameters for ***I_Na_*****.

**Name**	**Description**	**Published experimental data**		**Model parameter**	
		**a**	**b**	**2D**	**3D**
$V_{m}^{H}$ (mV)	Half-maximum activation voltage for *m* gate	–25.9 ± 1.2	–27.3 ± 1.3	–33.00	–33.00
*z*_*m*_	Movement of gate *m* gating particle	–	–	3.00	3.00
*k*_*m*_ (mV)	Slope factor: $k_{B}T∕(ez_{m}10^{-3})$	4.0 ± 0.3	–	≈ 8.5	≈ 8.5
*r*_*m*_ (ms^−1^)	Rate of activation for *m* gate	–	–	∞	∞
power_*m*_	# of independent *m* gates	1	1	3	3
$V_{h}^{H}$ (mV)	Half-maximum inactivation voltage for *h* gate	–38.9 ± 0.3	–48.1 ± 1.3	–42.14	–39.14
*z*_*h*_	Movement of gate *h* gating particle	–	–	1.11	5.20
*k*_*h*_ (mV)	Slope factor: $k_{B}T∕(ez_{h}10^{-3})$	4.8 ± 0.1	–	≈ 23.07	≈ 4.91
*r*_*h*_ (ms^−1^)	Rate of activation for *h* gate	–	–	0.2	0.2
γ_*h*_	Position of the energy barrier for *h* gate	–	–	0.38	0.38
power_*h*_	Number of independent *h* gates	1	1	1	1

*Comparison of published values derived from fits of voltage clamp data with values used in the models*.

^a^Olson et al. (2008);

*^b^Lin et al. (2009)*.

In the following we use *sb*_*def*_ as shown in Table 2 as a default parameter set to model Shab channels dynamics in order to relate the two dimensional analysis to the three dimensional results. As described below, the results for the three dimensional system agree with the characterization of the reduced two dimensional model.

### 3.2. General behavior of the two dimensional membrane model

In the following we keep ${\bar{g}}_{Na}$ constant and vary the dimensionless ratio ${\tilde{g}}_{sb}$ of ${\bar{g}}_{sb}$ and ${\bar{g}}_{Na}$. This serves as a measure for the Shab channel density, assuming that the maximal conductance is the product of the amount of channels in the membrane and the conductance of a single channel in the open state.

**Figure 3:**
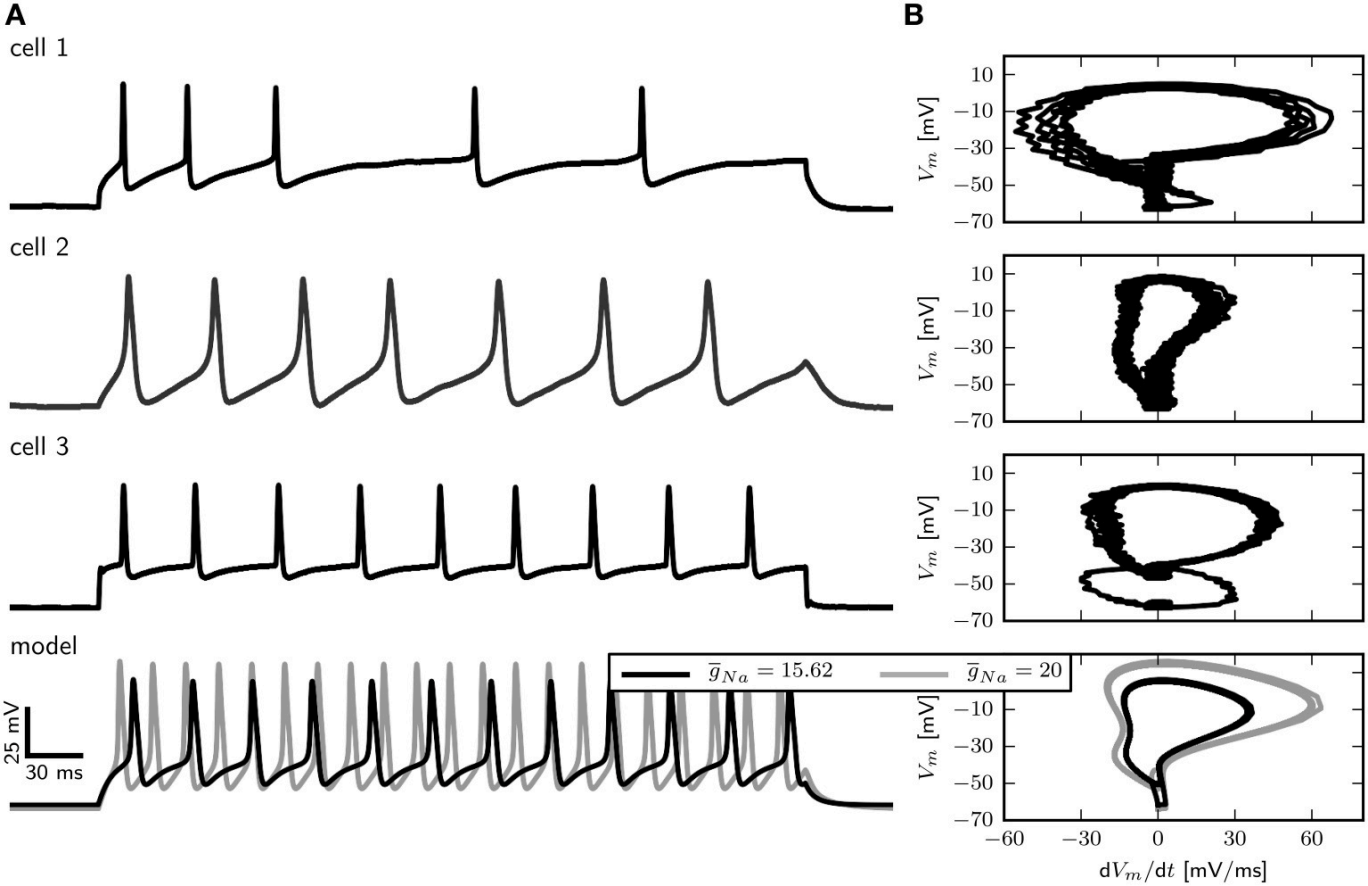
**Comparison of electrophysiological data and model behavior**. **(A)** Membrane potential in response to squared pulse current injections of different amplitudes in the model and three different cells. **(B)** Phase plots of electrophysiological recordings and the model in response to squared pulse current injection. Experimental methods provided by Ryglewski and Duch (2009).

With a ${\tilde{g}}_{sb}$ = 1.1347, the model resembles the basic features of *Drosophila* MN5 (Figure 3A) in response to steady current injection. Current pulses with amplitudes of the same order of magnitude as used in experiments cause the model to fire repetitively. However, compared to the majority of available recordings, the AP duration is longer and the firing frequency is higher (Figure 3A). It is likely that these features can be adjusted by adding A-type currents to the model. Shaker current is thought to adjust the spike shape (Peng and Wu, 2007), and Shal current is thought to decrease the firing frequency (Tsunoda and Salkoff, 1995a). Yet in some experimental recordings, the AP duration is even longer than in the model (Figure 3B). In contrast to our earlier models (Herrera-Valdez et al., 2013), in this model APs appear on elevated potentials for a wide range of parameters, including the default parameter set *sb*_*def*_. This is a consequence of the combination of slightly different values for γ_*b*_, *z*_*b*_, and *r*_*b*_.

In large part, the density of Na^+^ channels determines the maximal rate of change of the voltage (d*V_m_*∕d*t*) during the rising phase of an action potential. A phase-plane plot showing d*V_m_* ∕d*t* vs. *V*_*m*_ for three different recordings as well as the model is depicted in Figure 3B. The maximal value of d*V_m_*∕d*t* varies among cells and is between 55 and 20 mV/ms.

With a ${\bar{g}}_{Na}$ of 15.62 µS (Figure 3B right, black trace) d*V_m_*∕d*t* for the model takes a maximal value of 35 mV/ms, which is between the observed values. Increasing ${\bar{g}}_{Na}$ to 20 µS results in a maximal d*V_m_*∕d*t* of about 55 mV/ms during the rising phase, which corresponds to the largest observed values (Figure 3B, right, gray trace). In the following we show results for ${\bar{g}}_{Na}=\text{15.62}\;\text{µS}$. However, a model with ${\bar{g}}_{Na}=\text{20}\;\text{µS}$ in combination with a smaller ${\bar{g}}_{L}$ (0.01 µS for example) shows qualitatively similar behavior.

### 3.3. Shab channel density determines bifurcation type

**Figure 4:**
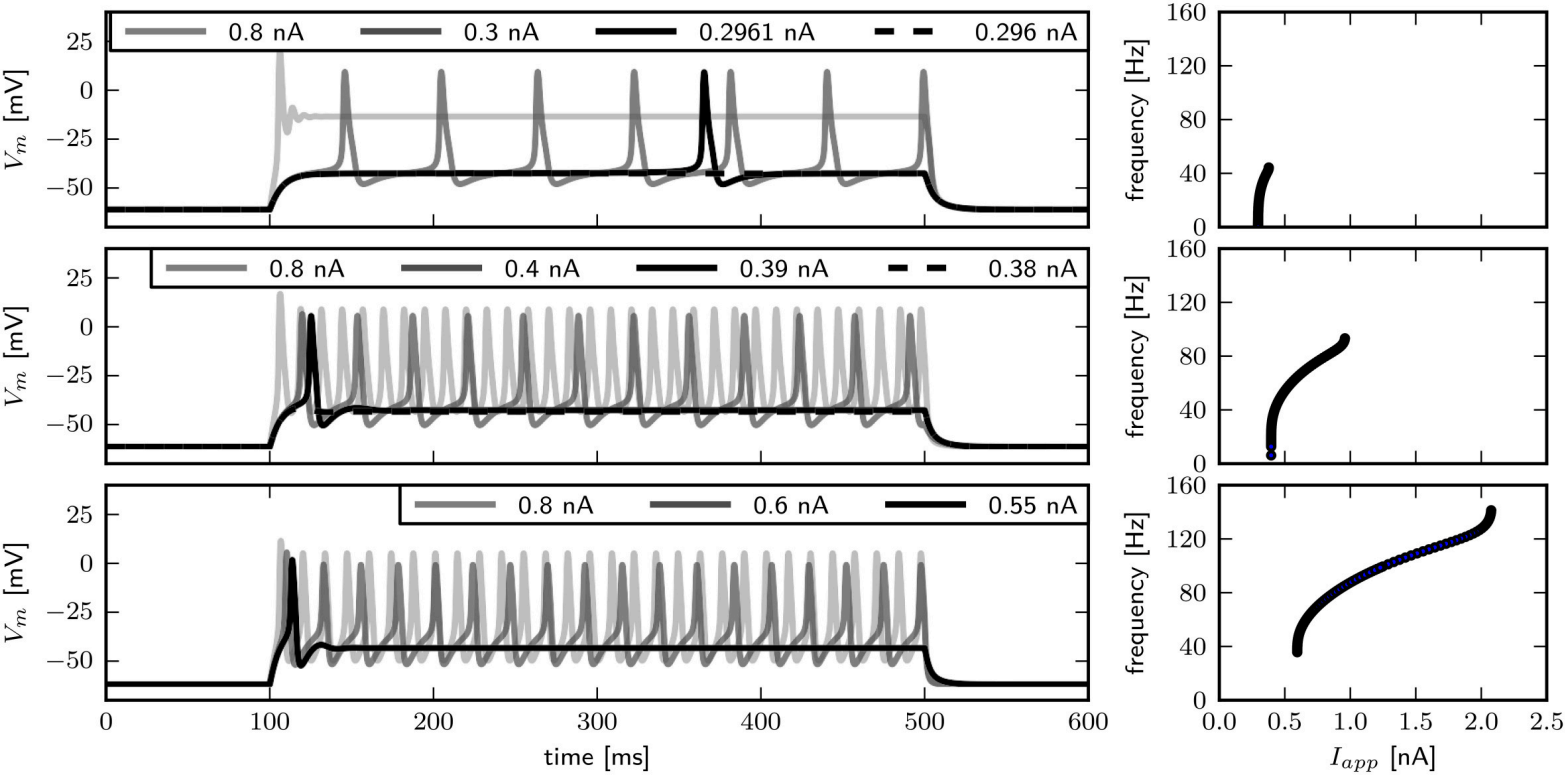
**Firing behavior for different relative Shab densities in the two dimensional model**. Responses to steady current injection (left) and *f* –*I* curves (right), from top to bottom ${\tilde{g}}_{sb}$: 0.6, 0.782, 1.1347 with *R*_*in*_ 70.2, 69.5, 65.4 and 59.4 MΩ. Note that changing the Shab densities affects *R*_*in*_.

In order to compare the model response to published experimental data, we examine the behavior of *V*_*m*_ over time (Figure 4). Visual inspection reveals that changing ${\tilde{g}}_{sb}$ has little influence on the spike shape. With ${\tilde{g}}_{sb}=0.6$, arbitrarily low firing frequencies with long delays to spike can be evoked, indicating type I dynamics. Current pulses with an amplitude of 0.8 nA elicit a single spike and subsequent low amplitude oscillations. Although the transition from rest to spiking occurs via different bifurcations, similar firing patterns are elicited with ${\tilde{g}}_{sb}$-values of 0.782 and 1.1347. In both cases a single spike can be elicited with small *I*_*app*_, while slightly increased stimulus amplitudes induce repetitive spiking with relatively high frequencies, indicating type II dynamics. Furthermore, the delay of spiking is small compared to observations when ${\tilde{g}}_{sb}=0.6$. In contrast to what was reported by Herrera-Valdez et al. (2013), the frequently observed pattern of a single spike with small and repetitive spiking with increased stimulus amplitudes in experiments can be generated with a system near a Hopf (Figure 4, bottom) as well as with a system near a saddle node bifurcation (Figure 4, middle). Note however that the range of current amplitudes where a single spike is elicited is narrow.

The two parameter bifurcation diagram (**Figure 6B**) displays the change of the locations of saddle-nodes and Hopf points in the *I*_*app*_ - ${\tilde{g}}_{sb}$ plane. Low levels of Shab density result in a saddle node bifurcation, and the saddle-nodes remain until ${\tilde{g}}_{sb}$ is about 1.2216, where the two branches collide at a cusp point. However, near ${\tilde{g}}_{sb}=0.7895$ and *I*_*app*_ = 0.4087 nA there is a Bogdanov–Takens bifurcation. At this point the saddle node curve meets a Hopf curve, and for higher values of ${\tilde{g}}_{sb}$, the fixed point loses stability at a Hopf point. This demonstrates that a non-monotonic *I*–*V*-curve does not necessarily indicate a loss of stability via a saddle node bifurcation. At ${\tilde{g}}_{sb}\approx 0.7$ the right branch of the Hopf curve crosses the right branch of the saddle node curve, meaning that the system has two stable fixed points for an increasing range of *I*_*app*_.

**Figure 5:**
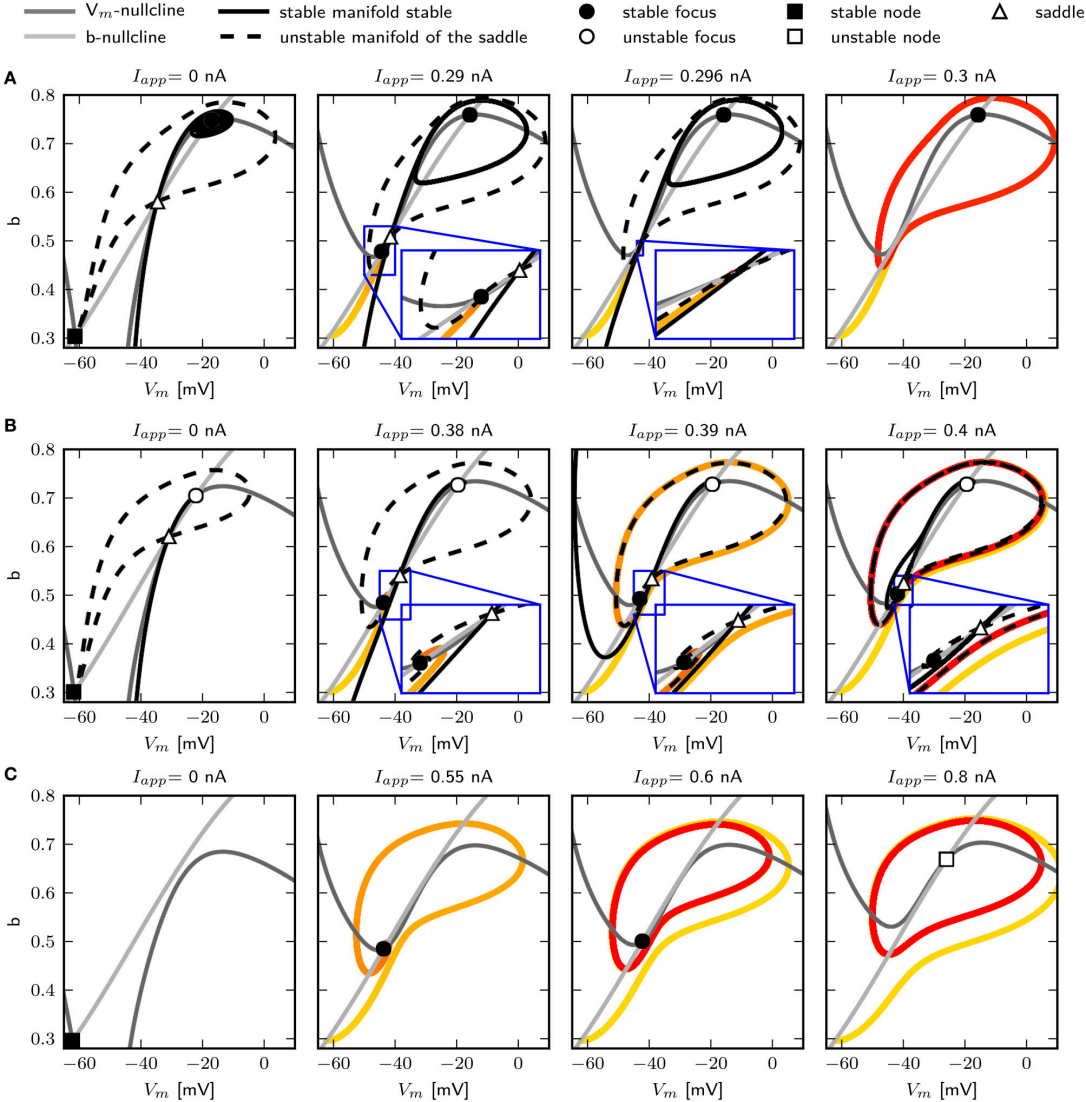
**Changes of fixed point types and trajectories with increased constant current injection in phase space**. ${\tilde{g}}_{sb}$ 0.6 **(A)**, 0.782 **(B)**, and 1.1347 **(C)**. Trajectories corresponding to initial conditions of the steady state without current injection are color coded from yellow (*t* = *t*_0_) to red (*t* = *t*_*max*_). Fixed points, unstable and stable manifolds of the saddle from left to right for in increasing *I*_*app*_ as indicated.

**Figure 6:**
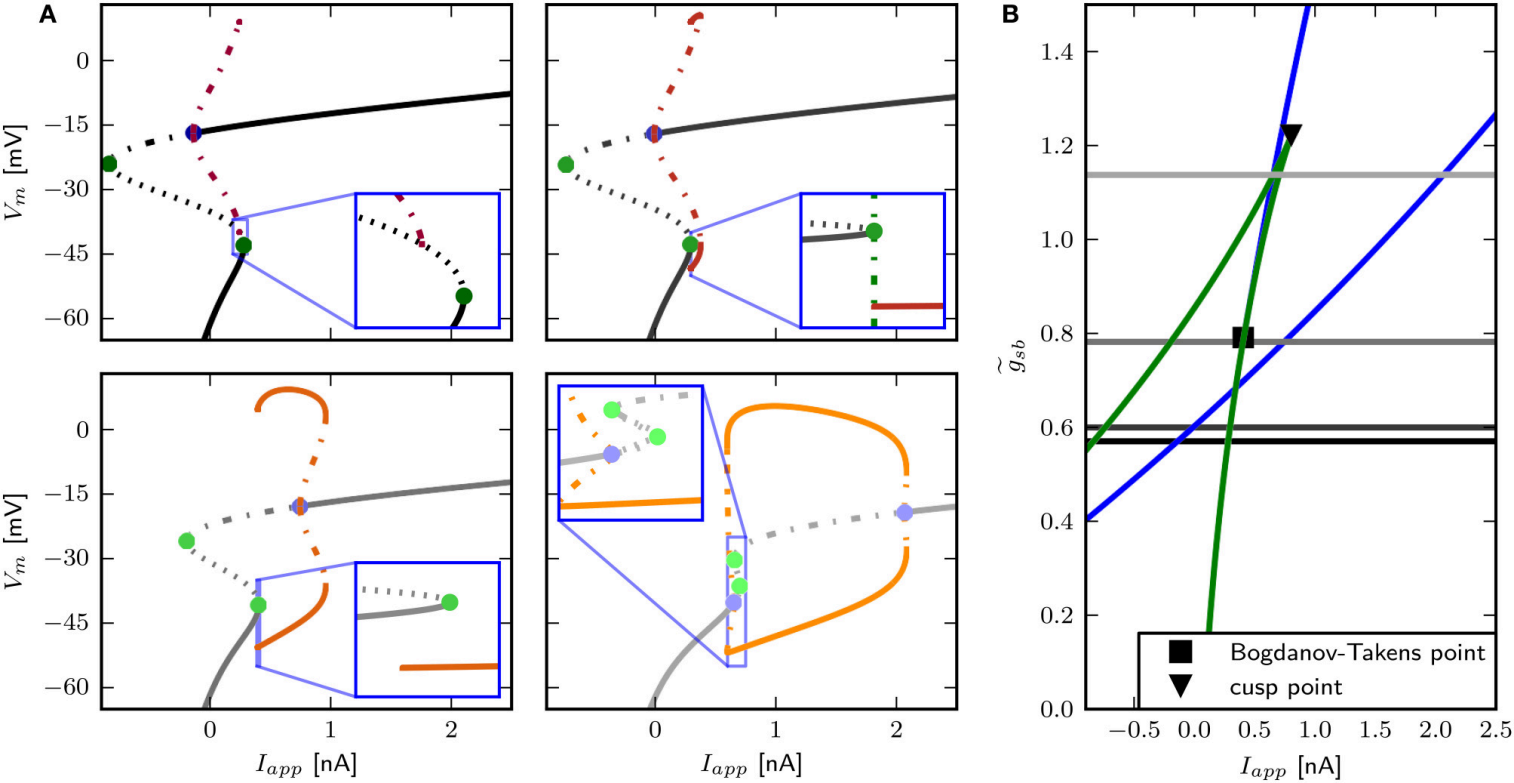
**Bifurcation structure for different relative Shab densities**. **(A)** One parameter bifurcation diagrams for ${\tilde{g}}_{sb}$: 0.57, 0.6, 0.782, 1.1347 (black to gray lines). Increasing ${\tilde{g}}_{sb}$ changes the transition from rest into spiking from a saddle node (green points) to a Hopf (blue points) bifurcation. Note that the stable branch continues outside of the shown region for negative values of *I*_*app*_. The minimum and maximum of periodic solutions are marked by red to orange lines for increasing ${\tilde{g}}_{sb}$. The lines style indicates the stability (solid: stable; dashed: unstable). **(B)** Two parameter bifurcation diagram showing the course of the Hopf (blue) and saddle node (green) curves in the *I*_*app*_ x ${\tilde{g}}_{sb}$ plane.

We use phase plane analysis to further investigate the characteristics of the system with three chosen ${\tilde{g}}_{sb}$-values, for which stable periodic solutions can be induced. The model is stimulated with constant current injections of different amplitudes. The amplitudes were chosen to be just below, at and just above the emergence of the limit cycle as determined by the bifurcation diagram of **Figure 6**. Figure 5 shows the phase plane indicating the fixed points and the stable and unstable manifolds of the neutral saddle, when they exist. Without or with small subthreshold *I*_*app*_, the unstable manifold connects the saddle node and the stable fix point to form a heteroclinic trajectory (Figures 5A,B, left). Letting ${\tilde{g}}_{sb}=0.6$, and injecting a current of 0.296 nA, the unstable saddle point and stable node coalesce and disappear. The trajectory becomes a homoclinic invariant circle and gives rise to a limit cycle with infinite period; that is, stability is lost via a SNIC bifurcation. When *I*_*app*_ is increased further, low frequency spiking is induced (Figure 5A, right).

Increasing ${\tilde{g}}_{sb}$ to 0.782, a single spike can be elicited with *I*_*app*_ = 0.39 nA. At this value the unstable manifolds still forms a heteroclinic orbit; however, the shape of the stable manifold changes so that it separates the initial values at rest and the stable fixed point (Figure 5B, middle). Hence a single spike is elicited because the trajectory must take a large excursion in order to approach the equilibrium. At *I*_*app*_ = 0.395 nA, one unstable manifold converges onto itself, and a limit cycle attractor appears (Figure 5B, right). The trajectory moves around the stable fixed point indicating a saddle big homoclinic bifurcation, where a stable and unstable manifold of a saddle point coincide and form a homoclinic orbit, and the two other branches lie inside the homoclinic orbit. The unstable manifold separates the stable fixed point and the initial conditions at rest, resulting in repetitive spiking. A saddle node bifurcation occurs for *I*_*app*_ above 0.4 nA.

When increasing ${\tilde{g}}_{sb}$ to 1.1347, stability is lost via a Hopf bifurcation (Figure 5C). With *I*_*app*_ = 0.55 nA, a single spike is elicited (Figure 5C, left). A stable limit cycle appears with *I*_*app*_ = 0.6 nA, while the fixed point remains stable (Figure 5C, middle). The fixed point becomes unstable when increasing *I*_*app*_ (Figure 5C, right).

We find that changing ${\tilde{g}}_{sb}$ results in different bifurcation types as the system undergoes a transition from rest to spiking in response to current injection (Figure 6) where low levels of Shab result in a saddle node bifurcation and high levels in a Hopf bifurcation. Figure 6A shows the bifurcation diagram for ${\tilde{g}}_{sb}$-values of 0.57, 0.6, 0.782, and 1.347. This diagram shows the local stability of the fixed points (black) of the system and indicates Hopf points (blue) and saddle nodes (green). In all cases there are saddle nodes, but for ${\tilde{g}}_{sb}=1.347$ the stability changes via a subcritical Hopf bifurcation at *I*_*app*_ = 0.653 nA. A stable limit cycle emerges at *I*_*app*_ = 0.595 nA, hence a stable fixed point and stable limit cycle coexist. The coexistence of a stable fixed point and a stable limit cycle is also given with ${\tilde{g}}_{sb}=0.782$. Yet, the stability of the fixed point is lost via a saddle node bifurcation at *I*_*app*_ = 0.4037 nA, while the stable limit cycle appears at *I*_*app*_ = 0.3958 nA via a global fold bifurcation of limit cycles (Figure 6A, bottom left panel inset). Decreasing ${\tilde{g}}_{sb}$ further to 0.6, the stable fixed point looses stability at *I*_*app*_ = 0.4037 nA and a stable limit cycle emerges (Figure 6A, top right panel inset), indicating a saddle node on invariant cycle bifurcation and hence type I excitability. The saddle node bifurcation is also present with ${\tilde{g}}_{sb}=0.57$, yet no stable limit cycle could be found. Instead, an unstable limit cycle appears at about *I*_*app*_ = 0.248 nA and remains unstable until it disappears at the Hopf bifurcation. The trajectories of the system will traverse the phase space toward the more depolarized stable fixed point, if the system is pushed beyond the bifurcation point.

### 3.4. Responses to time varying input

These different bifurcation types imply different phase response curves (PRCs) and different responses to periodic forcing. Therefore, the next step is to calculate the PRCs and analyze the model behavior for periodic current stimulation.

**Figure 7:**
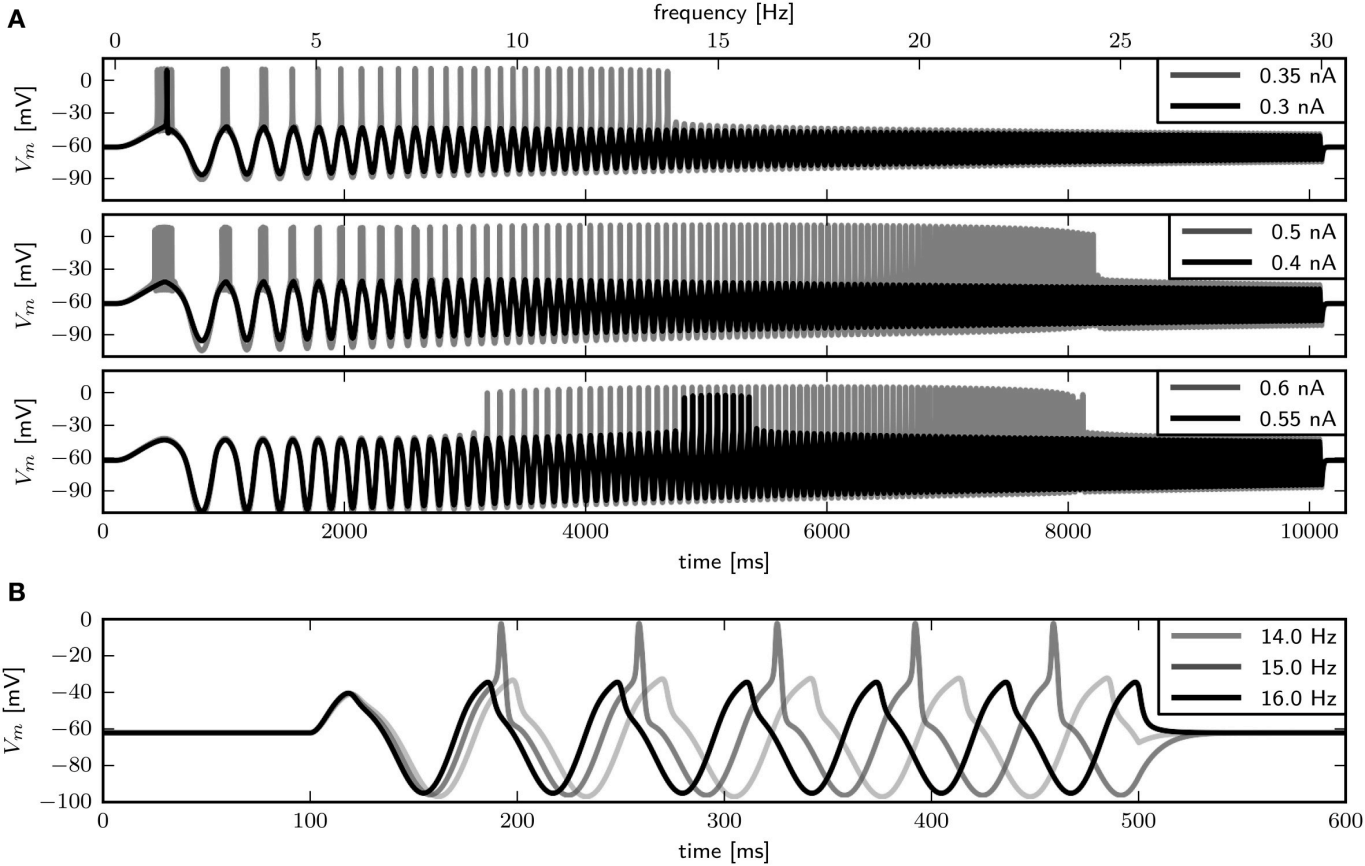
**Firing behavior in response to periodic forcing for different relative Shab densities in the two dimensional model**. **(A)** Responses to swept sine current injection, with frequencies from 0 to 30 Hz. From top to bottom ${\tilde{g}}_{sb}$: 0.6, 0.782, 1.1347. **(B)** Responses of the model with ${\tilde{g}}_{sb}$ = 1.1347 to sinusoidal forcing with three different frequencies.

We use periodic current injections with the shape of a sine wave with varying periods to assess the model response to periodic forcing and to find the preferred frequency of the model. The frequency sweeps from 0 to 30 Hz linearly over a time interval of 10 s. As expected, systems near a saddle node bifurcation with ${\tilde{g}}_{sb}$ of 0.6 or 0.782 displays integrator properties, behaving as a low-pass filter of the input current (Figure 7A, top and middle panel). In both cases the amplitude of *V*_*m*_ decreases with stimulus frequency. Current amplitudes above the respective bifurcation points are required to induce APs even at low frequencies. With larger amplitudes repetitive firing is induced at low frequencies and the spike count per cycle declines with increasing frequency. In contrast, with ${\tilde{g}}_{sb}=1.1347$, the model exhibits resonator properties, acting as a band-pass filter of input current. Stimulation with low amplitude swept sine current injection reveals that frequencies around 15 Hz elicit APs, while higher and lower frequencies only cause graded responses (Figure 7A, bottom panel). Upon increasing the stimulus amplitude, the frequency band that yields spiking becomes larger. Sinusoidal current injections confirm that with an amplitude of 0.55 nA a frequency of 15 Hz, but not 14 or 16 Hz results in the generation of APs (Figure 7B). In summary, while the response properties to constant current injection with ${\tilde{g}}_{sb}$ of 0.782 and 1.1347 are quite similar, the responses to periodic forcing are dramatically different.

How a rhythmically firing neuron responds to perturbations can be analyzed with the PRC, which provides a measure for the timing of the subsequent AP after a brief stimulus at a specific time during the spiking cycle. If a neuron spikes repetitively with a specific frequency with constant *I*_*app*_ or without a driving force, the state of the neuron can be expressed by a single phase variable. To obtain the phase, the time of a peak voltage equates to a phase of zero and subsequent times are divided by the spiking period. The PRC gives the phase shift induced by a perturbation as a function of the phase at which the perturbation occurs. Positive values indicate a phase advance, which means the next AP will be sooner, while negative values indicate a phase delay, which means the next AP will be later than without perturbation. PRCs that are mostly positive are called type I, whereas PRCs that have a positive and a negative component are classified as type II. In order to classify PRCs as type I or type II, Tateno and Robinson (2007) introduced a *r*-value, the ratio of maximal phase delay and advance, of 0.175.

**Figure 8:**
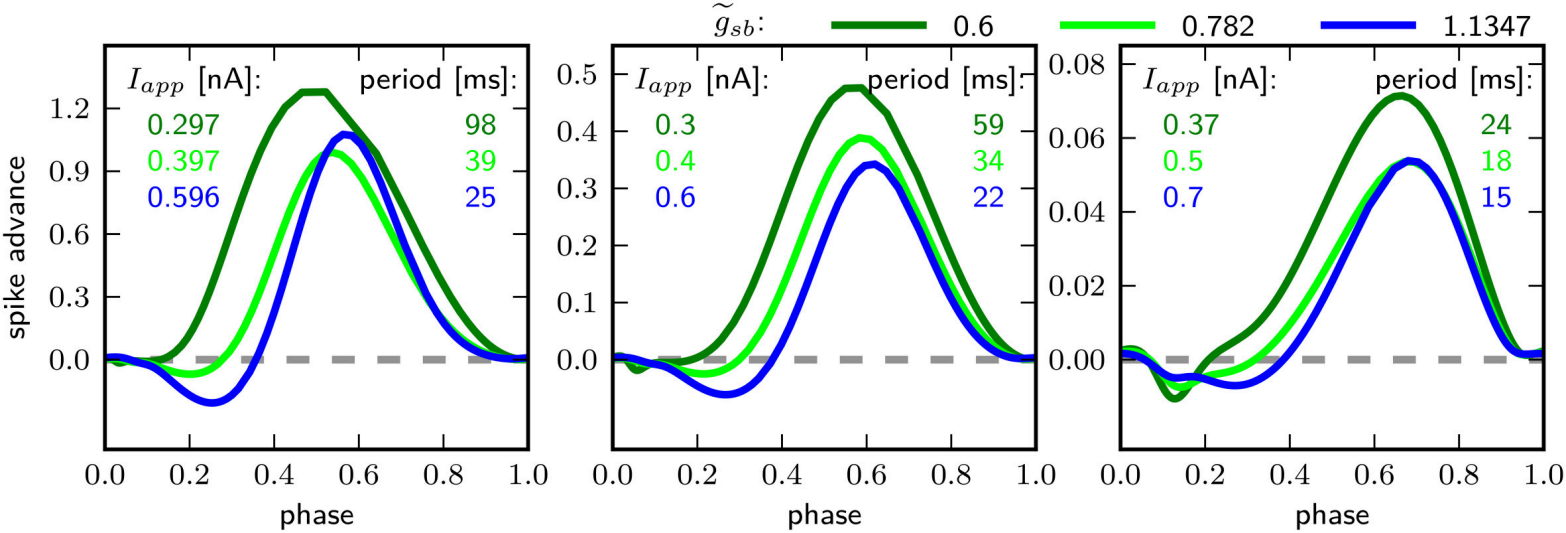
**Phase response curves for different relative Shab densities (indicated by colors) and from left to right for increasing ***I_app_***, as indicated**.

Figure 8 shows the PRCs of the model for the different indicated ${\tilde{g}}_{sb}$-values. For each parameterization of ${\tilde{g}}_{sb}$ three different values for *I*_*app*_ were used, which starts close to the firing threshold and is increased subsequently. With ${\tilde{g}}_{sb}=0.6$ the PRC can be classified as type I, with *r*-values of 0.01, 0.04, and 0.15 for *I*_*app*_ 0.297, 0.3, and 0.37nA. By increasing ${\tilde{g}}_{sb}$ to 0.782, the PRC has small negative values for small phase values, but the *r*-values with 0.067, 0.064, and 0.14 for *I*_*app*_ 0.397, 0.4, and 0.5 still leads to a classification as type I. With a further increase of ${\tilde{g}}_{sb}$ to 1.1347, the negative values in the early phase increase, for small baseline *I*_*app*_ of 0.596 and 0.6 nA the PRC is type II, with *r*-value of 0.192 and 0.179 bigger than 0.175. However, with a baseline *I*_*app*_ of 0.7 nA the *r*-value is 0.130. As can be seen by the bifurcation diagram in Figure 6, although the stable fixed point loses stability via a Hopf bifurcation, there is also a saddle node bifurcation near *I*_*app*_ = 0.7 nA, which might influence the vector field in a way that the model exhibits a type I PRC. For higher baseline *I*_*app*_ the *r*-value increases and exhibits a PRC classified as type II (not shown). Also when increasing ${\tilde{g}}_{sb}$ to 1.5 the PRC is classified type II for all probed stimulus amplitudes that elicit repetitive spiking (not shown).

The PRC corresponding to all three ${\tilde{g}}_{sb}$-values have in common that they are relatively insensitive to perturbations at the beginning and the end of the cycle shortly before and after the peak of the AP. Further, the maximum of the PRC shifts to the right with higher baseline stimulation.

### 3.5. Small parameter changes induce delay to first spike

**Figure 9:**
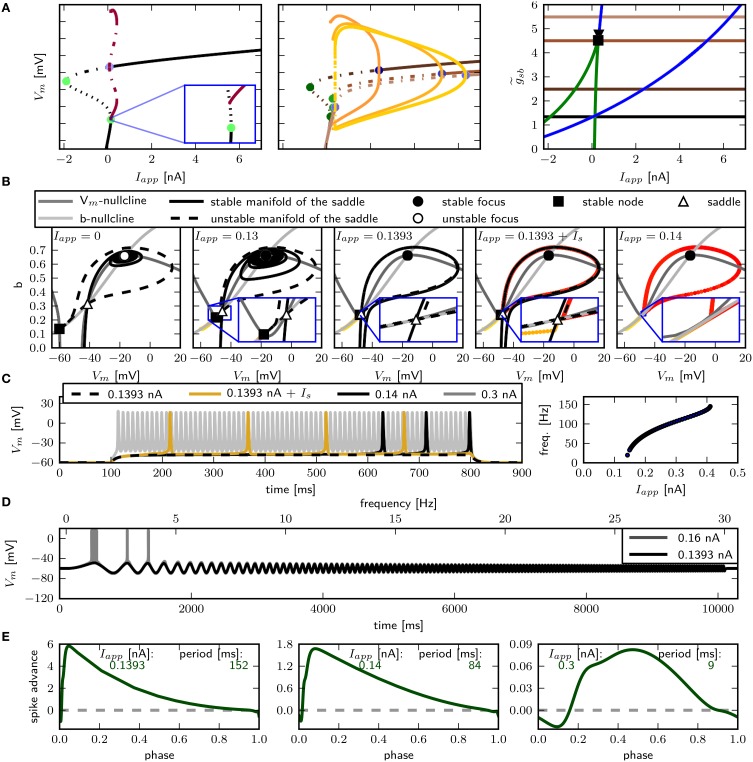
**Saddle small homoclinic bifurcation with changed Shab parameters**. **(A)** Right: Bifurcation diagram shows, that with ${\tilde{g}}_{sb}$ = 1.35 a stable limit cycle (dark red) coincides with the curve of saddle nodes (black dots), while the stable fixed point vanishes via a saddle node bifurcation at a bigger *I*_*app*_. Middle: Increasing ${\tilde{g}}_{sb}$ (dark to lighter brown and orange for fixed points and periodic solutions) changes the transition from rest into spiking from a saddle node (green points) to a Hopf (blue points) bifurcation. Dashed and solid lines mark stability (solid: stable; dashed unstable). Left: Two parameter bifurcation diagram indicating the location of Hopf points (blue) and saddle nodes (green) as well as the Bogdanov–Takens (square) and cusp (triangle) points with respect to ${\tilde{g}}_{sb}$. **(B)** Changes of fixed point types and trajectories for different *I*_*app*_ in phase space. From left to right *I*_*app*_ increases as indicated. Trajectories corresponding to initial conditions of the steady state without current injection are color coded from yellow (*t* = *t*_0_) to red (*t* = *t*_*max*_). **(C)** Left: Responses to constant current injection. The stimulus inducing the response shown in yellow exhibits an additional short current pulse (*I*_*s*_) with 30 ms and 0.01 nA at *t* = 150 ms. Right: *f* –*I* curve. **(D)** Responses to swept sine current injection, with frequencies from 0 to 30 Hz. **(E)** PRCs for increasing *I*_*app*_, as indicated.

The only firing profile that could not be reproduced by changing ${\tilde{g}}_{sb}$ is an onset with a long delay and subsequent higher frequency firing. Usually a saddle small homoclinic bifurcation is responsible for such behavior (Izhikevich, 2007). We explored the parameter space further in order to find a regime where the system undergoes this bifurcation. We set *z*_*b*_ to a constant value of 1.5 in order to obtain a better description of the Na^+^ channel inactivation (see Section 3.1). Then we performed the fitting routine, resulting in the parameter set *sb*_*z*_ (see Table 2) with which the desired firing profile can be reproduced (Figure 9C). The bifurcation diagram for ${\tilde{g}}_{sb}=1.35$ reveals that at *I*_*app*_ ≈ 0.1392 nA the minimum on the periodic orbit collides with the saddle node, and the coexisting stable fixed point is at a smaller *V*_*m*_ (Figure 9A). By increasing ${\tilde{g}}_{sb}$, the system exhibits the same bifurcation types as with the default parameters *sb*_*def*_. With this set of parameters ${\tilde{g}}_{sb}$ must be much higher in order to produce the various firing patterns than with the previously used parameters.

Phase plane analysis confirms that the underlying bifurcation when ${\tilde{g}}_{sb}=1.35$ is indeed a saddle small homoclinic bifurcation (Figure 9B), where one stable and one unstable manifold of the saddle node collide and form a homoclinic orbit; the remaining two manifolds lie outside the homoclinic orbit. At low values of *I*_*app*_, one of the unstable manifolds connects to the less depolarized stable fixed point, traversing around the more depolarized stable fixed point as well as around the stable manifold that emerges from the unstable periodic orbit around that fixed point (Figure 9B, left). Increasing *I*_*app*_ beyond the bifurcation point, the unstable manifold for the saddle node converges onto itself and the stable manifold is now on the outside of the resulting limit cycle (Figure 9B, middle right). The saddle point and the stable node both remain, hence the model is bistable. A constant current pulse does not induce repetitive spiking, because the state of the system is not pushed beyond the stable manifold. Applying an additional short pulse can push the system onto a trajectory that converges onto the periodic orbit (Figure 9B, right and Figure 9C, yellow line). However, the range of *I*_*app*_-values where this occurs is extremely narrow, suggesting that it is not likely to be observed experimentally.

As expected for a system near a saddle node bifurcation, the model exhibits integrator properties, as revealed by swept sinusoidal current injection (Figure 9D). The PRC is biphasic (Figure 9E) with an *r*-value above 0.175, but differs from those shown in Figure 8. A perturbation shortly before, during and after the peak of an AP results in a delay of the subsequent AP. For small *I*_*app*_, the region where a perturbation causes the strongest phase advance is shortly after the peak of the AP and much earlier during the spiking cycle at a phase of approximately 0.1. Increasing *I*_*app*_ to 0.3 nA, the peak of the phase advance shifts to the right, and the PRC has more resemblance to the ones shown in Figure 8.

### 3.6. Impact of leak conductance

**Figure 10:**
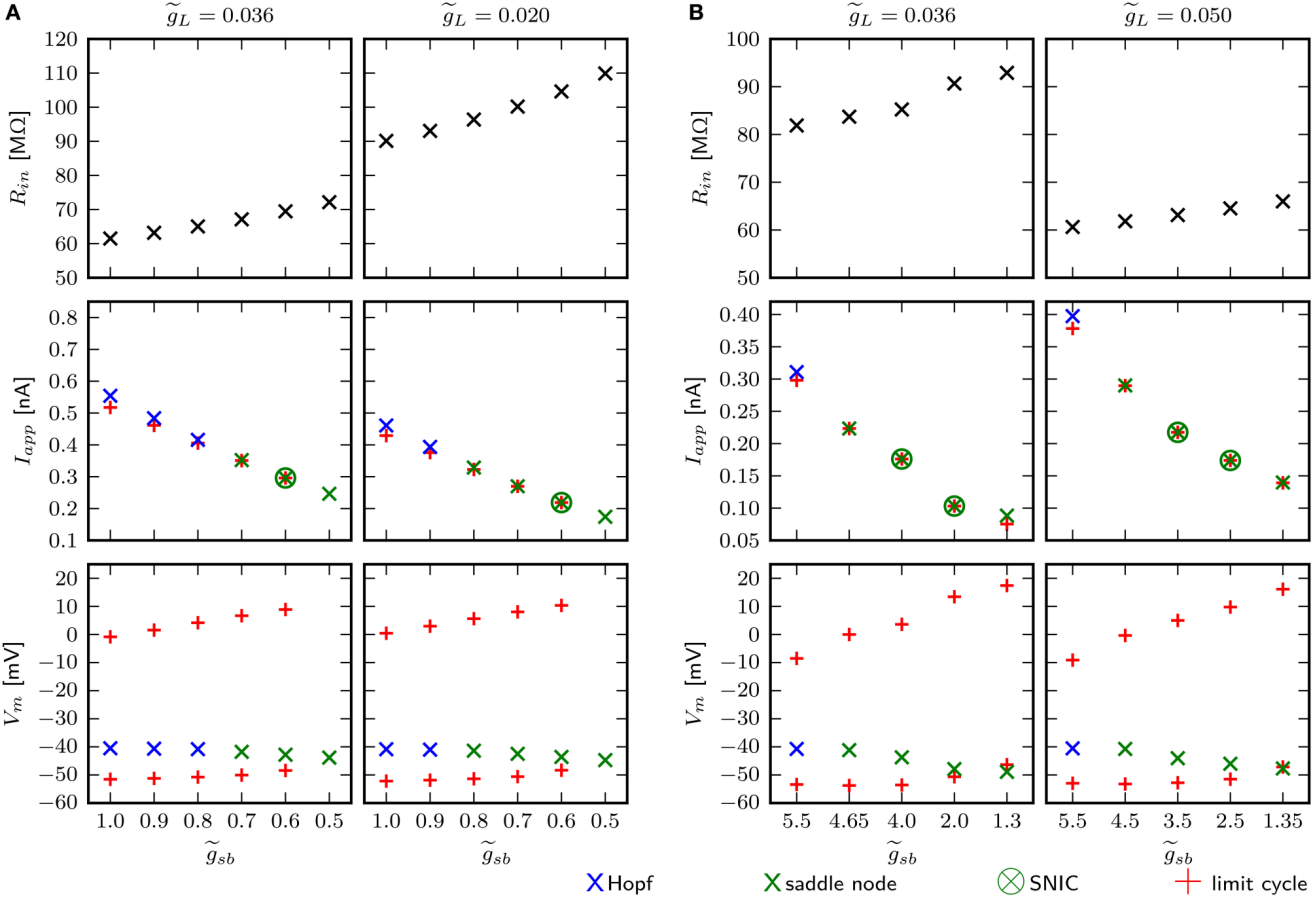
**Small changes of ${\tilde{g}}_{L}$ adjust for *R*_*in*_, but have no influence on bifurcation types the system can exhibit**. Different aspects of the model behavior as function of ${\tilde{g}}_{sb}$ for two different ${\tilde{g}}_{L}$. In panels **(A)** and **(B)** the default and changed Shab channel models are used, respectively. Top panels show *R*_*in*_ measured with small current injections. Middle panels show the minimal amplitudes of *I*_*app*_ for which a stable limit cycle emerges (red pluses) and the fixed point loses stability (blue and green crosses). Bottom panels show *V*_*m*_ of the fixed points near the bifurcation (blue and green crosses) and the minimum and maximum of the limit cycles (red pluses).

An estimate for the input resistance (*R*_*in*_) is obtained by applying Ohm's law to the steady state voltage response for small *I*_*app*_. With the chosen value of 0.036 for ${\tilde{g}}_{L}$, depending on the Shab density, *R*_*in*_ is between 59.4 and 70.2 MΩ (Figure 10A, top left). ${\tilde{g}}_{L}$ is the dimensionless ratio of ${\bar{g}}_{L}$ and ${\bar{g}}_{Na}$. This is below the experimentally measured *R*_*in*_ in MN5 of 97±31 MΩ (Duch et al., 2008). However, the amplitude of *I*_*app*_ for which the stable fixed point loses stability and/or a stable limit cycle emerges for small ${\tilde{g}}_{sb}$ is below 0.4 nA (Figure 10A, middle left), which is the typical amplitude that induces repetitive spiking in MN5. For higher ${\tilde{g}}_{sb}$, on the other hand, the spiking threshold is too high. *R*_*in*_ and the inversely related amplitude of *I*_*app*_ that elicits APs can be adjusted by changing ${\tilde{g}}_{L}$. We decrease ${\tilde{g}}_{L}$ to 0.02 and investigate whether this changes the bifurcations the system exhibits. As before, ${\tilde{g}}_{sb}$ was set to different values and the fixed points, periodic solutions and bifurcations that occur as *I*_*app*_ is changed are determined using numerical continuation. To resolve whether a limit cycle occurs via a SNIC or homoclinic bifurcation, we compare the minimal *I*_*app*_ for which a periodic solution was found with *I*_*app*_ of the saddle node bifurcation (Figures 10A,B, middle). Equal amplitudes of *I*_*app*_ indicate a SNIC, otherwise a saddle homoclinic bifurcation is indicated. To further distinguish between a small and big homoclinic bifurcation, the value of *V*_*m*_ at the fixed point and at the minimum of the periodic orbit are compared (Figures 10A,B, bottom). This analysis reveals that with increased ${\tilde{g}}_{L}$, the same bifurcation types can be elicited by changing ${\tilde{g}}_{sb}$. The change between bifurcations may occur at different ${\tilde{g}}_{sb}$ levels; i.e. with ${\tilde{g}}_{sb}=0.8$ stability is lost via Hopf bifurcation when ${\tilde{g}}_{L}=0.036$, while it is lost via a saddle node bifurcation when ${\tilde{g}}_{L}=0.02$. However, increasing ${\tilde{g}}_{L}$ leads to spiking onset in response to smaller *I*_*app*_ (Figure 10A, middle) and decreasing ${\tilde{g}}_{L}$ further ultimately results in an unstable steady state without current injection, with small ${\tilde{g}}_{sb}$. Therefore, a loss of stability via a Hopf bifurcation is the only possibility for a model that is quiescent with *I*_*app*_ = 0 nA. Also, it cannot be excluded that there exists a ${\tilde{g}}_{sb}$-value for which the model exhibits a saddle small homoclinic bifurcation resulting in the appearance of a stable limit cycle, but this value was not found.

The chosen default value for the leak conductance is a compromise in the attempt to match sensitivity to current injection and input resistance with experimental data. The apparent missmatch might be a consequence of the fact that the model incorporates only two channel types. With the changed Shab parameters, the default value of ${\tilde{g}}_{L}$ with 0.036 results in higher *R*_*in*_, but in turn, the bifurcations occur at rather small values of *I*_*app*_. Therefore, ${\tilde{g}}_{L}$ is increased to 0.05. Figure 10B demonstrates that the change in ${\tilde{g}}_{L}$ is not the cause of the saddle small homoclinic bifurcation resulting in the appearance of a stable limit cycle.

### 3.7. Independent Na^+^ channel inactivation

To further investigate whether the changed bifurcation structure found with the new parameter set *sb*_*z*_ is a consequence of altering the activation of the Shab current, the inactivation of the Na^+^ current or whether it is necessary to alter both channels, we reintroduce an additional variable representing the gating of the Na^+^ channel inactivation. This allows for a better fit of the Na^+^ inactivation as demonstrated in Section 3.1. Increasing the gating charge results in a good match to the inactivation curve derived by Olson et al. (2008) (Figure 2B). However, the time constants are not available from these data. Using the same values as before, the model exhibits APs with a peak of -10 mV and the maximal d*V_m_*∕d*t* is below 20 mV/ms. This is adjusted by increasing ${\bar{g}}_{Na}$ to 20 µS and decreasing ${\tilde{g}}_{L}$ to 0.02.

**Figure 11:**
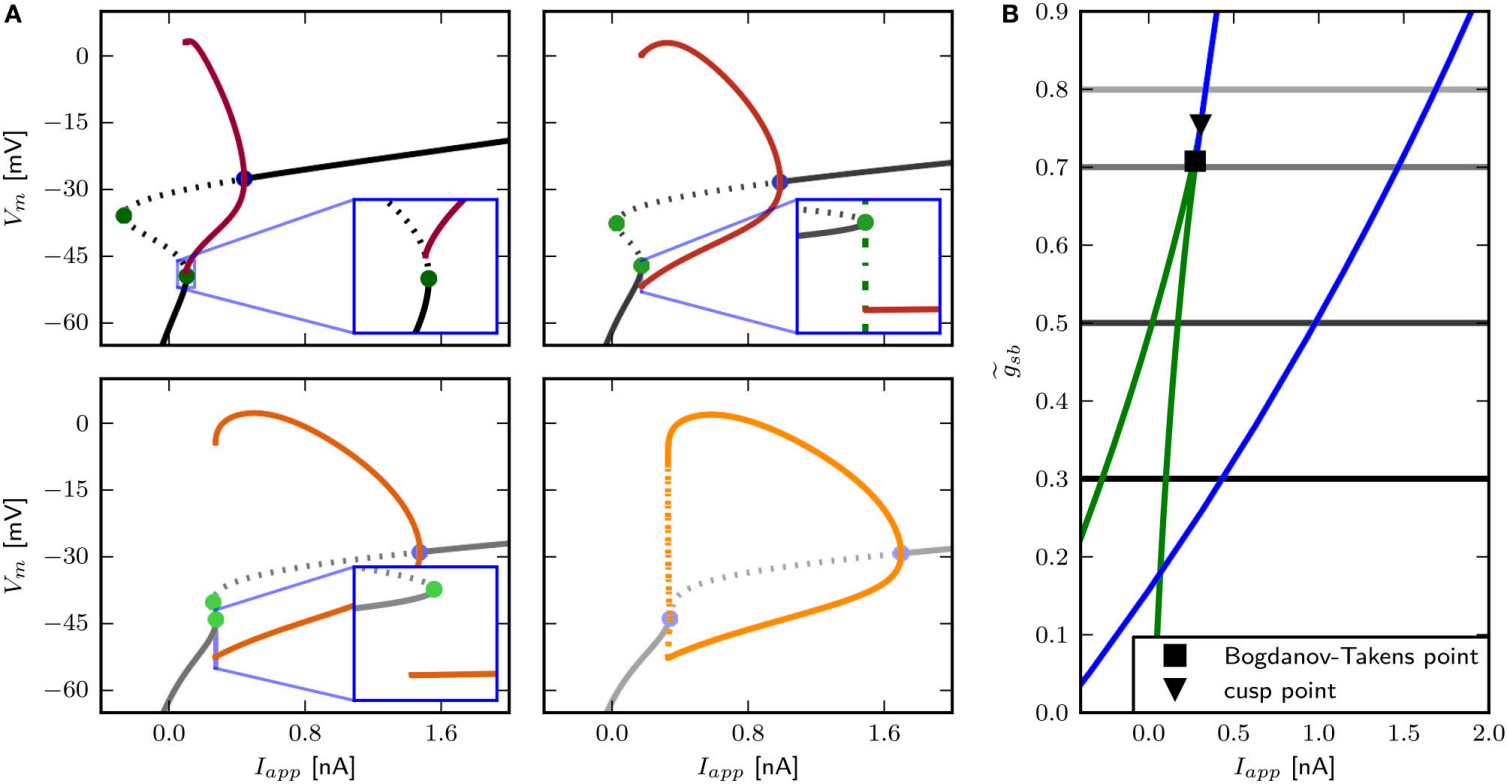
**Bifurcation structure for different Shab channel densities in the three dimensional model**. **(A)** Bifurcation diagram shows that increasing ${\tilde{g}}_{sb}$ changes the transition from rest into spiking from a saddle node bifurcation (blue points) to a Hopf bifurcation (green points). Fixed points are drawn in black to gray and the minimum and maximum of the periodic solutions shown in red to yellow for ${\tilde{g}}_{sb}$: 0.3, 0.5, 0.7 and 0.8. Dashed and solid lines mark stability (solid: stable; dashed unstable). **(B)** Two parameter bifurcation diagram indicating the location of Hopf points (blue) and saddle nodes (green) with respect to ${\tilde{g}}_{sb}$.

**Figure 12:**
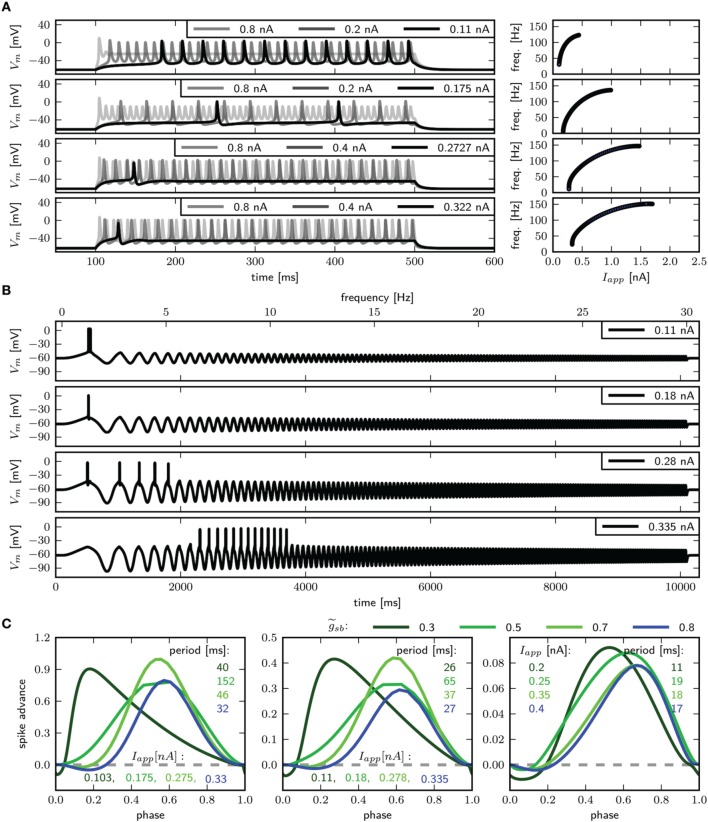
**Firing behavior and PRC for different Shab channel densities in the three dimensional model**. Responses to **(A)** steady and **(B)** swept sine (frequencies 0–30 Hz) current injection for (from top to bottom) ${\tilde{g}}_{sb}$: 0.3, 0.5, 0.7, 0.8; with *R*_*in*_ 94.5, 82.7, 75.1 and 72.1 MΩ. **(C)** PRCs for increasing *I*_*app*_, as indicated.

With the three dimensional formulation, similarly to the two dimensional model, high Shab densities result in a Hopf bifurcation, while low densities result in a saddle node bifurcation (Figure 11). Firing patterns similar to those observed with the two dimensional model are generated. With very low ${\tilde{g}}_{sb}$ levels, the branch of the minimas of the periodic solution coincides with the unstable branch of saddle points (Figure 11A, upper left panel and inset). The minimum of the periodic orbit is therefore above the stable fixed point, which coexists at the stimulus amplitude where the limit cycle emerges. In the two dimensional model, this was due to a saddle small homoclinic bifurcation (see Section 3.5). As in the two dimensional case, the membrane responds with a long delay to first spike and subsequent higher firing frequency to small square pulse current injections (Figure 12A, first panel), the model acts as low-pass filter (Figure 12B, first panel), and the PRC is biphasic displaying a phase delay shortly before and after an AP. A phase advance occurs at < 0.1 and > 0.9 of the phase; systems with other bifurcation structures display only weak responsiveness to perturbations at this time within the cycle (Figure 12C). Increasing ${\tilde{g}}_{sb}$ to 0.5, stability is lost via a SNIC bifurcation (Figure 11A, lower left panel and inset). The model exhibits infinitely low frequency and a delay to first spike with the same duration as the following ISI (Figure 12A second panel), low-pass filter (Figure 12B, second panel) properties, and a type I PRC (Figure 12C). With ${\tilde{g}}_{sb}=0.7$ the stable fixed point still loses stability via a saddle node bifurcation; however, a periodic orbit emerges before that point (Figure 11A), lower left panel and inset), with the minimal value of *V*_*m*_ smaller than for the stable fixed point. In the two dimensional model, phase plane analysis revealed that this is due to a saddle big homoclinic bifurcation. With ${\tilde{g}}_{sb}$-values of 0.7 and 0.8, a single spike is induced for sufficiently low current stimulation. With higher current injection, a shorter delay to first spike than the following ISI (Figure 12A third and fourth panel) is seen. The PRCs are similar as well (Figure 12C), but the model shows resonator properties only when stability is lost via a Hopf bifurcation with ${\tilde{g}}_{sb}=0.8$ (Figure 12B).

In summary, with this model, smaller current injection leads to repetitive spiking, and the changes between bifurcation types occur for smaller ${\tilde{g}}_{sb}$. Furthermore, increasing the stimulus amplitude results in spikes with smaller amplitude, and the transition out of spiking at high current levels occurs via a subcritical Hopf bifurcation. As in the two dimensional model with the changed parameters for the Shab channels, the three dimensional model displays four types of bifurcations when varying the Shab channel density by a factor of 4. This demonstrates that the additional bifurcation due to changed parameters in the two dimensional model (see Section 3.5) can be achieved by simply adjusting the Na^+^ channel inactivation kinetics.

## 4. Discussion

In this study, we develop a two dimensional excitable membrane model with currents based on biophysical features of *Drosophila* Shab and DmNa_v_29 channels. This model was used to assess which features of experimentally observed firing behavior can be produced by varying only the Shab channel density. Using phase plane and bifurcation analysis, the model was investigated and tuned to reproduce experimentally observed firing patterns. The bifurcation types the models undergo as current is injected were assessed as channel densities varied using stability analysis. The two dimensional model was then extended to a three dimensional model in order to include independent Na^+^ inactivation.

In previous studies, we investigated the influence of A-type channels on *Drosophila* MN5 firing behavior and found that, in accordance with experimental results, Shal and Shaker can influence the firing patterns and bifurcation structure of a neuron differently (Herrera-Valdez et al., 2009, 2010). It is known that regenerative currents like the persistent Na^+^ and A-type K^+^ current support aggregator properties connected to SNIC bifurcations, the type I PRCs, and class I *f* –*I* curves. On the other hand, for example the delayed rectifier K^+^ current evokes resonator properties associated with a Hopf bifurcation, type II PRCs, and the class II *f* –*I* curves (Hutcheon and Yarom, 2000; Ermentrout et al., 2001). However, in order to determine for which behaviors the incorporation of K^+^ channels with voltage dependent inactivation kinetics are necessary, it is useful to know whether those properties can be induced without those channels.

We find that a wide array of firing patterns can be generated with only two currents. Specifically, properties like the long delay to first spike and slow firing frequencies, that were thought to be unique features requiring A-type channels (Choi et al., 2004; Schaefer et al., 2010; Ping et al., 2011), can be induced in our model.

### 4.1. Minimal excitable membrane model

We developed a minimal model consisting of one regenerative current that amplifies changes, one linear leak current, and one restorative current that counteracts changes of *V*_*m*_. The regenerative current is a Na^+^ current; the restorative current is a delayed rectifier K^+^ current based on channels encoded by *Shab*. In *Drosophila* there are only four genes, *Shaw, Shab, Shal*, and *Shaker*, that encode voltage gated K^+^ channels. Shab channels were chosen because it has been shown that they provide delayed rectification in *Drosophila* and are required to induce repetitive firing, which also can be inferred by the different dynamics of the channels. Shaw channels have a very low voltage sensitivity and mediate a leak current. Shaker and Shal channels mediate transient A-type current, where their steady state activation curves are more hyperpolarized than the one associated with Shab channels, and they express fast inactivation kinetics. This indicates a role in regulating spike initiation and repetitive firing patterns rather than a major contribution to inducing repetitive firing. Additionally, Shaker mutants display broader APs and higher firing frequencies, while Shal has been reported to increase the spiking threshold and to induce a delay to first spike (Choi et al., 2004; Ping et al., 2011).

In the model, ${\bar{g}}_{Na}$ was assigned by comparing the wave forms in response to current injection of the *in situ* measured *V*_*m*_ at the soma of MN5 and the model. We did not aim to reproduce the shape of the APs exactly, since we only incorporate a small subset of channels in our models, and it is likely that the additional channels influence the shape measured in MN5. Instead the maximal d*V_m_*∕d*t* was compared. However, it is not clear whether the depolarization observed at the soma results from passive spread of APs, whether there is an additional spike generating zone close by or at the soma, or whether active properties at the soma boost the AP generated at the spike initiation zone (SIZ). When the SIZ is far away from the soma it is possible that the shape of APs observed at the soma is attenuated and broader than at the SIZ due to the filtering properties of passive structures. The maximal d*V_m_*∕d*t* measured from recordings of MN5 is small compared to values reported in other preparations, where it is about 100 and 300 mV/ms in neurons of the cat visual cortex *in vivo* and *in vitro* (Naundorf et al., 2006) or about 200 mV/ms in ganglion cells from the tiger salamander (Fohlmeister and Miller, 1997). This means that the maximal d*V_m_*∕d*t* at the SIZ, and thus ${\bar{g}}_{Na}$ may be higher.

### 4.2. Relation of channel density and firing patterns

Our results demonstrate that small changes in ${\tilde{g}}_{sb}$ are sufficient to produce a variety of different firing patterns that relate to those observed experimentally. The differences can be linked to a change in the underlying mathematical structure of the system, yielding qualitatively different transitions between rest and spiking. The differences in observed firing behaviors of MN5 *in situ* indicate that the neuron might be close to a bifurcation with co-dimension greater than one, where such a change of the bifurcation type with respect to *I*_*app*_ occurs. However, MNs are required to exhibit reliable outputs during behavior since they are the final processing station, and alternating input-output behavior cannot be adjusted by network properties. Therefore, the observed differences could be due to different modulatory states that tune the neurons' output for specific needs. For example, biogenic amines like octopamine and tyramine influence the take-off likelihood and flight maintenance significantly (Brembs et al., 2007). Further, MN5 exhibits tonic firing during flight, only single APs and no doublets or triplets are observed; during male courtship song, pulse firing is shown. Although this could be due to different input, the different firing patterns may also be supported by differences in the intrinsic excitability and thus the modulatory state.

In the model, low Shab channel concentrations induce behavior thought to be induced by A-type channels. However, at small ${\tilde{g}}_{sb}$, the range of stimulus amplitudes for which a stable periodic solution exists is rather narrow. In the three dimensional model and the model with altered channels kinetics, the AP amplitude increases drastically with increased current.

Some experimentally observed features of the firing patterns could not be reproduced well by this model. Further channels may be necessary to support some behavior combinations like integrator properties together with a higher input resistance and a higher firing threshold. Furthermore, adaptation could not be generated, since it requires a third time scale, and therefore a further variable (Guckenheimer et al., 1997).

Bistability occurs in the model for a very specific combination of constant stimulus amplitude and timing of pulse stimulation. Although bistability has not been reported for experimental studies, the model results suggest that it is unlikely this phenomenon would be observed. If observed, the neuron might be rejected and assessed as being not intact. Further, a long delay to first spike with subsequent ISI of similar duration, as typical for a system close to a SNIC bifurcation, was not reported as an observed firing pattern in Herrera-Valdez et al. (2013). However, this firing pattern was recorded at least once in *eag* and *Shaker* double knock downs, as shown in the Figure 2C of Duch et al. (2008). It must be noted that the quantification of firing patterns of wild type MN5 as reported in Duch et al. (2008) and Herrera-Valdez et al. (2013) is different, indicating that perhaps the recording conditions changed between these sets of experiments.

### 4.3. Variability in ion channel density

It has been shown that ion channel expression, as well as the maximal conductances of ion currents in neurons, can be extremely variable. *Shab* mRNA in MNs of the crustacean stomatogastric nervous system vary two- to four-fold (Schulz et al., 2006, 2007) and four-fold in gastric mill neurons. In *Drosophila* MN5, voltage clamp recordings reveal that the peak of the total K^+^ current can vary by a factor of about four (Ryglewski and Duch, 2009). In our models a variation of up to four-fold is sufficient to produce the different firing patterns and bifurcation structures. However, it has also been shown that the expression patterns of certain ion channel combinations are coordinated, where the specific combinations depend on the cell type. In gastric mill neurons Na^+^ and Shab channels are correlated, but in all other investigated cells, the mRNA abundance of the genes encoding these two channels is independent (Schulz et al., 2007). In a recent review article Marder (2011) pointed out that these findings indicate the need “to measure as many system components as possible within an individual” and that the process of fitting and combining independently obtained and averaged experimental data is unlikely to reproduce realistic model behavior. Especially in the small *Drosophila*, it is hard to measure a variety of system parameters simultaneously. Similarly, pharmacological manipulations may suffer from low specificity of drugs (Greenwood and Leblanc, 2007). These issues can potentially lead to incorrect conclusions about the role of a certain ion channel, however, the use of computational models can help resolve these issues, by allowing for controlled and independent variation of specific parameters to understand the impact of biological variations and allow for an examination of the impact of parameters that are not experimentally separable.

### 4.4. Influence of phasic input on output behavior

During flight, the firing frequency of the MNs is modulated simultaneously (Levine and Wyman, 1973) and studies support the idea that the firing frequency is an inherent property of the neurons (Harcombe and Wyman, 1977), which changes when the required power output is changed (Gordon and Dickinson, 2006). Together with the observation that two MNs never fire in a time window of about 5 ms, it was concluded that all MNs receive common excitatory input and that the MNs interact reciprocally (Harcombe and Wyman, 1977). In other words, the common excitatory input induces periodic spiking of the units, and the reciprocal interactions cause a perturbation of the period depending on the phase when the input is received. An analysis of how a neuron responds to phasic input while firing repetitively is measured with PRCs. The claim of Harcombe and Wyman (1977) and Koenig and Ikeda (1983) that there is a strong interaction if one MN fires simultaneously or shortly before another MN, requires a PRC with large phase shifts at the very beginning and end of the period, as observed in models close to a saddle small homoclinic circle. Those models also display a long delay to first spike with shorter ISIs to constant current injection, a firing pattern that is often observed in neurons at larval stage, but rather seldom in adult MN5s. However, in our model, this pattern corresponds to current injections very close to the spiking threshold. Therefore, it could also mean that this pattern manifests at stimulus amplitudes that fall between the probed current steps.

In the two dimensional model with the default channel kinetics, a delay to first spike could not be reproduced. We have shown with the electro-diffusion model, that this does not require the introduction of a further variable (Herrera-Valdez et al., 2013) rather a change of *z*_*b*_ in combination with a low ${\tilde{g}}_{sb}$-value elicits this behavior. In the model investigated here, a simple change of *z*_*b*_ is not sufficient to produce this profile. However, it is produced by models with moderate changes in more than one parameter and more importantly, after incorporating independent Na^+^ inactivation dynamics.

### 4.5. Comparison to other excitable membrane models

Various minimal conductance based models have been employed in previous studies in order to analyze the electrical behavior of neurons. For example Prescott et al. (2008) employed a two dimensional model based on the Morris–Lecar model (Morris and Lecar, 1981; Rinzel and Ermentrout, 1998) and showed that all three types of excitability could be reproduced. However, here we show that a neuron model based on more realistic ion channel mechanisms also can exhibit all three types of firing behavior. Zeberg et al. (2010) used models in which diverse K^+^ currents were combined as one recovery variable and found that the variation of channel densities can switch the model from resonator to integrator. This study demonstrates that incorporating only one delayed rectifier K^+^ channel in the model and varying its density is sufficient to induce this switch.

In an earlier modeling effort, we contributed to the development of an electro-diffusion based model for MN5 membrane potential dynamics (Herrera-Valdez et al., 2013). However, this previous model falls short in reproducing the Na^+^ channel kinetics, and it is not clear whether the theoretical improvement of the electro-diffusion based model justifies the accompanied increase in complexity. In general, an advantage of the electo-diffusion approach is that the maximal currents can be fitted directly to electrophysiological data. However, electrophysiological data are often preprocessed under the assumption of a linear current–voltage relationship, for example when leak currents are subtracted from the traces. Unlike the conductance-based model that we present here, the previous electro-diffusion based model also fails to generate spikes on elevated potentials as observed experimentally. However, this *in situ* observation can also emerge when the recording site is far away from the SIZ, which cannot be addressed with a minimal model approach employed by Herrera-Valdez (2012) and here.

In another recent study, a model of *Drosophila* third-instar larval motoneuron was developed in order to investigate the impact of alternative splicing of Na^+^ channels (Lin et al., 2012). In that model the Na^+^ channels have activation kinetics as in the classical Hodgkin–Huxley model formalism, and the time constant is based on electrophysiological recordings in embryonic *Drosophila* motoneuron (O'Dowd and Aldrich, 1988). The measured Na^+^ current is possibly a combination of currents mediated by different splice variants of the *DmNa*_*v*_*29* gene that are specific to embryonic neurons. Here, we use instantaneous activation of Na^+^ channels for two reasons: first due to the advantage of reducing the model to only two dimensions and second because there are no data for the time course of specific splice variants available.

In conclusion, we find that a wide array of firing patterns like those seen in experimental studies can be generated with only two currents, and we show that a neuron model based on realistic ion channel mechanisms can exhibit all three types of firing behavior. This study demonstrates that incorporating only one delayed rectifier K^+^ channel in the model and increasing its density is sufficient to induce a switch from type I excitability to type II excitability, with a corresponding switch from integrator to resonator properties. Additionally, properties like the long delay to first spike and slow firing frequencies, that were thought to be unique features requiring A-type channels (Choi et al., 2004; Schaefer et al., 2010; Ping et al., 2011), can be induced in our model. As described above, additional channel types are known to be present and play a role in *Drosophila* motoneuron dynamics, leading to many possible, more complex alternatives for obtaining similar results. It is plausible that having more channels that in different combinations produce a similar behavior can add to the robustness of the system and provide a way to adjust for natural variability. Certainly, the minimal model described here can guide our understanding of the interactions among channel types in this important model system.

## Funding

SB and SC were supported in part by the National Science Foundation (NSF IIS 0613404). Support for SB was also provided by the Interdisciplinary Graduate Program in Neuroscience at Arizona State University.

### Conflict of interest statement

The authors declare that the research was conducted in the absence of any commercial or financial relationships that could be construed as a potential conflict of interest.
